# Magnesium Attenuates Renal Senescence and Fibrosis With Reduced DNA Damage Response and H3K4me3 Enrichment at the *p16^INK4a^* Promoter

**DOI:** 10.1096/fj.202504665RR

**Published:** 2026-08-14

**Authors:** Makoto Matsubara, Kensuke Sasaki, Shigehiro Doi, Takeshi Ike, Maria Yoshida, Akira Takahashi, Yosuke Osaki, Naoki Ishiuchi, Aiko Okubo, Yujiro Maeoka, Takuto Chiba, Ayumu Nakashima, Takao Masaki

**Affiliations:** ^1^ Department of Nephrology Hiroshima University Hospital Hiroshima Japan; ^2^ Division of Nephrology, Department of Pediatrics University of Pittsburgh School of Medicine Pittsburgh Pennsylvania USA; ^3^ Department of Nephrology, Graduate School of Medicine University of Yamanashi Yamanashi Japan

**Keywords:** cGAS‐STING pathway, DNA damage, epigenetics, inflammation, kidney injury, magnesium, senescence

## Abstract

Renal fibrosis is a final pathway leading to end‐stage renal disease, with cellular senescence contributing to fibrosis and inflammation. Magnesium ions (Mg^2+^) are implicated in DNA stabilization and epigenetic regulation. In this study, we hypothesized that Mg^2+^ ameliorates renal fibrosis in association with reduced DNA damage responses and injury‐induced cellular senescence, along with altered histone H3K4 trimethylation. To test this, we used murine models of radiation‐induced organ injury and renal ischemia–reperfusion injury (IRI), along with primary cultured mouse renal proximal tubular cells. Mice received intraperitoneal MgSO_4_ (600 mg/kg) before radiation or IRI, with repeated dosing (300 mg/kg) after IRI. Cultured cells were treated with 6.4 mM MgSO_4_. We demonstrated that Mg^2+^ provided protection against radiation injury and reduced radiation‐induced DNA damage markers in renal cells both in vitro and in vivo. Furthermore, Mg^2+^ suppressed IRI‐induced morphological alterations, DNA damage, and cellular senescence in the kidneys, while inhibiting renal inflammation and cGAS‐STING pathway activation, along with attenuation of renal fibrosis in IRI model mice. Consistent with these findings, a reduction in the expression of pro‐inflammatory cytokines and fibrosis‐related genes was observed. Finally, Mg^2+^ was associated with decreased p16^INK4a^ transcription and reduced H3K4 trimethylation levels at its promoter in primary renal tubular cells. Our findings suggest that Mg^2+^ alleviates renal DNA damage while protecting against inflammation and fibrosis with accompanying epigenetic modulation. Although clinically relevant pharmacological Mg^2+^ dosing and therapeutic applicability require further investigation, these insights may inform therapeutic strategies targeting fibrosis and senescence‐related kidney disease.

## Graphical abstract

Magnesium (Mg^2+^) attenuates renal injury in ischemia–reperfusion and radiation‐induced kidney injury models in association with reduced DNA damage response markers, cellular senescence, inflammation, and fibrosis. Mg^2+^ treatment is associated with reduced H3K4me3 enrichment at the p16^INK4a^ promoter, attenuation of senescence‐associated pathways, and reduced SASP‐related inflammatory responses and cGAS–STING pathway activation. These findings suggest that reduced DNA damage‐associated cellular stress and altered epigenetic regulation may contribute to the renoprotective effects of Mg^2+^.

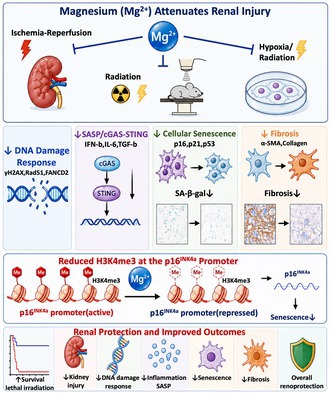

## 1 Introduction

Chronic kidney disease (CKD) affects approximately 850 million people worldwide (8%–15% of the global population) and is now recognized as a major global health issue owing to its strong association with serious complications, including cardiovascular disease and all‐cause mortality [1, 2]. Epidemiological studies have consistently shown that the prevalence of CKD increases with aging, underscoring the close relationship between cellular senescence and impaired kidney function [3]. Importantly, regardless of the underlying cause of CKD, tubulointerstitial fibrosis in the kidney represents a decisive and common final pathway that promotes progression to end‐stage renal disease [4, 5]. Pathologically, CKD is characterized by excessive accumulation of extracellular matrix proteins in the renal interstitium, an aberrant fibrotic response that leads to tissue scarring and progressive organ dysfunction [6, 7]. However, the precise mechanisms driving CKD progression remain incompletely understood, with current therapeutic options largely limited to interventions that merely delay its progression [8, 9].

The DNA damage response is a critical event that leads to cellular senescence. Unrepaired DNA damage accumulates within cells, impairing cellular function, accelerating senescence, and contributing to the development of various diseases [10]. Cellular senescence involves permanent cell cycle arrest, characterized by a sustained inability of cells to proliferate. This occurs due to the convergent upregulation of the cyclin‐dependent kinase inhibitors p16^INK4a^ and/or p21^WAF1/CIP1^, leading to cell cycle arrest at the G1/S checkpoint and the induction of senescent phenotypes [11]. In recent years, the accumulation of senescent cells has been increasingly recognized as a key contributor to fibrotic diseases [12]. Renal senescence, which is induced by aging and various stressors, including ischemia–reperfusion injury (IRI), has been reported by our group and others to promote renal fibrosis and inflammation [13, 14]. The cyclic GMP‐AMP synthase (cGAS)‐stimulator of interferon genes (STING) pathway is a crucial component of the innate immune system. This pathway is activated by the recognition of double‐stranded DNA (e.g., mitochondrial DNA) in various cell types, including innate immune cells, epithelial cells, and endothelial cells, and functions as a key mediator of inflammation in tissue injury conditions [15, 16]. Importantly, recent studies have demonstrated that cGAS, a cytosolic DNA sensor that activates innate immunity, is essential for the induction of cellular senescence [17], and that STING‐mediated inflammation contributes to the development of renal fibrosis [18]. However, the regulatory mechanisms that suppress cGAS‐STING‐mediated senescence and subsequent renal fibrogenesis remain largely unknown.

Magnesium ion (Mg^2+^) is an abundant cation in the intracellular environment, playing a pivotal regulatory role in various physiological processes [19, 20, 21], partly attributable to its ability to store energy in the form of Mg^2+^–ATP complexes [22]. Beyond the general role of cations in neutralizing the negative charge of DNA, Mg^2+^ binds specifically to negatively charged oxygen and nitrogen atoms within polynucleotide chains, forming essential components of the tertiary DNA structure that promote chromatin compaction and contribute to DNA stabilization [19, 22, 23, 24]. Furthermore, accumulating evidence suggests that Mg^2+^ has anti‐inflammatory effects [25, 26], antioxidant properties [27, 28], and anti‐apoptotic activity [29] across various experimental systems, including the kidney. However, the organ‐protective effects of Mg^2+^ against renal fibrosis and the precise molecular mechanisms involved have yet to be fully elucidated.

Epigenetics involves mechanisms for regulating gene expression without alterations in the DNA sequence and includes processes such as DNA methylation, histone modifications, and microRNA‐mediated interference [30, 31]. The role of epigenetic regulation in senescence‐associated fibrosis has been reported by our group and others [13, 32, 33, 34]. Interestingly, Mg^2+^ may also function as an epigenetic modulator by influencing chromatin structure [21, 35, 36].

We hypothesized that Mg^2+^ attenuates renal fibrosis in association with reduced DNA damage‐associated cellular stress, cGAS‐STING pathway activation, and cellular senescence, along with altered histone H3K4 trimethylation (H3K4me3). To test this hypothesis, we utilized IRI model mice with radiation‐induced kidney injury and mouse‐derived primary cultured renal cells to investigate the role of Mg^2+^ in epigenetic regulation during DNA damage and cellular senescence, as well as its contribution to renal fibrosis. This study aimed to provide fundamental insights into the interplay among senescence, fibrosis, and epigenetic modifications, and to build a body of evidence supporting the protective effects of Mg^2+^ against DNA damage and renal fibrosis, potentially opening new avenues for therapeutic intervention.

## 2 Methods

### 2.1 Animals

Male C57BL/6J mice (age: 8–9 weeks; body weight: 20–25 g, RRID: MGI:3028467) were purchased from Charles River Laboratories Japan (Yokohama, Japan). The mice were housed in a light‐ and temperature‐controlled environment in the Laboratory Animal Center of Hiroshima University with ad libitum access to food and water. All animal procedures were approved by the Institutional Animal Care and Use Committee of Hiroshima University (approval no. A24‐23) and conducted in accordance with the NIH Guidelines for the Care and Use of Laboratory Animals and the ARRIVE guidelines.

### 2.2 Animal Treatment With MgSO_4_

For the renal IRI model, mice received an i.p. injection of magnesium sulfate (MgSO_4_, 600 mg/kg; 137‐12335; Fujifilm Wako, Tokyo, Japan) [37, 38, 39] 30 min prior to surgery. The MgSO_4_ dose was selected based on preliminary optimization experiments and prior experimental studies [38, 40]. After IRI induction, additional MgSO_4_ was administered by i.p. injection at 300 mg/kg every 12 h. Mice were euthanized on either Day 7 or Day 21 post‐IRI for subsequent analyses. For lethal dose radiation injury, mice received an i.p. injection of a total dose of 600 mg/kg MgSO_4_ at 30 min prior to irradiation. The control group was treated with the same volume of normal saline. To evaluate systemic Mg^2+^ exposure under the experimental dosing conditions used in this study, serum Mg^2+^ concentrations were measured at baseline and 1, 2, and 6 h after MgSO_4_ administration. Hemolyzed samples were excluded from analysis.

### 2.3 Radiation Injury Model in Mice

Mice were individually placed into 50‐mL plastic conical tubes. Tubes were arranged concentrically on the platform of an X‐ray generator (MBR‐1520R‐3; Hitachi Power Solutions, Ibaraki, Japan), and 8 Gy of radiation was delivered during platform rotation. In the lethal dose model, no shielding was used, and the mice were monitored for survival. In the renal injury model, the entire body except for the kidneys was shielded with a 2‐mm‐thick lead sheath, and the mice were euthanized 30 min postirradiation for kidney collection.

### 2.4 Cell Culture

NRK‐49F cells (RRID: CVCL_2144) and NRK‐52E (RRID: CVCL_0468) cells were obtained from the American Type Culture Collection (Manassas, VA, USA) and were cultured in DMEM (08458‐16; Nacalai Tesque, Kyoto, Japan) supplemented with 5% FBS (172012; Nichirei Bioscience, Tokyo, Japan) and 100 U/mL penicillin with 100 μg/mL streptomycin (09367–34; Nacalai Tesque). Cultures were maintained at 37°C in a humidified atmosphere with 5% CO_2_.

### 2.5 Mg^2+^ Administration and Radiation Exposure in Cell Cultures

NRK‐49F and NRK‐52E cells were cultured in 10‐cm dishes using standard DMEM, which contains 0.8 mM MgSO_4_. For higher magnesium conditions, MgSO_4_ was added to DMEM to a concentration of 6.4 mM. At 1 h prior to irradiation, the culture medium was replaced with DMEM containing either 0.8 mM or 6.4 mM MgSO_4_. The dishes were then arranged in a concentric circle on the rotating platform of a radiation device (Hitachi Power Solutions, Japan) and exposed to a single dose of 1 Gy [41]. Cells were collected 30 min after irradiation and subjected to subsequent in vitro analyses. The in vitro concentration (6.4 mM MgSO_4_) was selected based on preliminary optimization studies to provide sufficient Mg^2+^ exposure for mechanistic evaluation of DNA damage responses and senescence‐associated pathways [42].

### 2.6 Western Blot Analysis

Western blotting was performed as previously described [43, 44, 45], using the following antibodies: anti‐phospho‐H2AX (γH2AX) (#9718; Cell Signaling Technology, Danvers, MA, USA, RRID: AB_2118009), anti‐Rad51 (#8875; Cell Signaling Technology, RRID: AB_2721109), anti‐FANCD2 (ab108928; Abcam, Cambridge, UK, RRID: AB_10862535), anti‐p16^INK4a^ (ab51243; Abcam, RRID: AB_2059963), anti‐p21^WAF1/CIP1^ (ab109199; Abcam, RRID: AB_10861151), anti‐ac‐p53 (ab321819; Abcam), anti‐p53 (ab26; Abcam, RRID: AB_303198), anti‐p‐Rb (#9308; Cell Signaling Technology, RRID: AB_331472), anti‐total‐Rb (#9313; Cell Signaling Technology, RRID: AB_1904119), anti‐cGAS (#31659; Cell Signaling Technology, RRID: AB_2799008), anti‐STING (#13647; Cell Signaling Technology, RRID: AB_2732796), anti‐phospho‐TBK1 (#5483; Cell Signaling Technology, RRID: AB_10693472), anti‐TBK1 (#3504; Cell Signaling Technology, RRID: AB_2255663), anti‐α‐SMA (A2547; Sigma‐Aldrich, St. Louis, MO, USA, RRID: AB_476701), anti‐collagen type I (NBP1‐30054; Novus Biologicals, Littleton, CO, USA, RRID: AB_1968486), and anti‐GAPDH (G8795; Sigma‐Aldrich, RRID: AB_1078991). The secondary antibodies were horseradish peroxidase‐conjugated goat anti‐rabbit and goat anti‐mouse immunoglobulins (Dako, Agilent, Santa Clara, CA, USA). The intensity of each band was quantified using ImageJ software (version 1.53k; National Institutes of Health, Bethesda, MD, USA, RRID: SCR_003070).

### 2.7 Renal IRI Procedure

IRI was performed as previously described [13, 46]. Briefly, mice were anesthetized and then subjected to unilateral IRI with left renal pedicle clamping for 35 min using Micro Serrefines (18052‐02; Fine Science Tools, Foster City, CA). For sham operations, mice underwent the same surgical procedure without clamping of the renal pedicle. During the procedure, body temperature was maintained at 37°C using an external heating source. Mice were euthanized by cardiac puncture under deep anesthesia at 7 or 21 days post‐IRI.

### 2.8 Histology and Immunohistochemistry

Histological and immunohistochemical staining procedures were performed as previously described [47, 48]. Briefly, formalin‐fixed, paraffin‐embedded tissues were sectioned at 4 μm and subjected to staining with hematoxylin–eosin (HE) (109249 and 109844; Sigma‐Aldrich) and Masson's trichrome (MT) (HT15‐1KT; Sigma‐Aldrich), in accordance with the manufacturer's protocols. Quantification of the HE‐stained cells and fibrotic MT‐positive area was performed based on distinctive density patterns observed in five randomly selected image fields, analyzed using ImageJ software (NIH, Bethesda, MD, RRID: SCR_003070). For immunohistochemical staining, the following primary antibodies were used: anti‐α‐SMA (A2547; Sigma‐Aldrich, RRID: AB_476701), anti‐p16^INK4a^ (ab51243; Abcam, RRID: AB_2059963), anti‐p21^WAF1/CIP1^ (ab188224; Abcam, RRID: AB_2734729), anti‐collagen type I (2150‐1908; Bio‐Rad, Hercules, CA, USA, RRID: AB_620498), and anti‐F4/80 (#70076; Cell Signaling Technology, RRID: AB_2799771). Based on the distinctive density and color of immunostaining, we quantified the number and position of stained cells, as well as the area of positive staining observed in five randomly selected image fields using ImageJ.

### 2.9 Injury Score

Tubular injury was scored by colleagues in pathology who were unaware of the treatment status of the mice. Cortical ×400 fields were scored as follows: 0, no injury; 1, 1%–25%; 2, 26%–50%; 3, 51%–75%; and 4, 76%–100%. Tubular injury was defined as protein cast formation, tubular dilatation, tubular cell swelling, vacuolization, or tubular cell degeneration.

### 2.10 Quantitative Real‐Time PCR

RNA extraction and quantitative real‐time PCR (qPCR) were performed as previously described [34, 43]. Briefly, total RNA from kidneys was isolated using TRIzol reagent (Thermo Fisher Scientific, Waltham, MA, USA). A High‐Capacity cDNA Reverse Transcription Kit (Applied Biosystems, Foster City, CA, USA) was used to synthesize cDNA from equal amounts of total RNA from each sample. qPCR was performed using a CFX96 Real‐Time System (Bio‐Rad). For TaqMan‐based qPCR analyses, reactions were performed using TaqMan Universal PCR Master Mix (Applied Biosystems) and the following TaqMan Gene Expression Assays: *Il1b* (Mm00434228_m1), *Il6* (Mm00446190_m1), *Acta2* (Mm00725412_s1), *Col1a1* (Mm00801666_g1), *Col3a1* (Mm00802300_m1), and *Gapdh* (Mm99999915_g1). Gene expression levels were normalized to *Gapdh*. For SYBR Green–based qPCR analyses, reactions were performed using SYBR Green Master Mix (Bio‐Rad) with gene‐specific primers for *Tnf*, *Ccl2*, *Cxcl1*, *Cxcl2*, *Tgfb1*, *Ccn2*, *Serpine1, Ifnb1, Isg15, Mx1, Cxcl10, Irf7*, and *Ifit1*. Primer sequences are listed in Table S1. Melt curve analysis confirmed amplification specificity. Gene expression was normalized to *Gapdh or Rps18*, as specified in the corresponding figure legends, and analyzed using the 2^−ΔΔCt^ method.

### 2.11 Senescence‐Associated β‐Galactosidase Staining

Senescence‐associated β‐galactosidase (SA‐β‐gal) staining (#9860; Cell Signaling Technology) was performed according to the manufacturer's instructions to detect senescent cells. Frozen kidney tissue sections (4 μm thick) were fixed using the fixative solution provided with the kit for 10 min at room temperature, washed with PBS, and then incubated for 24 h at 37°C (without CO_2_) in freshly prepared SA‐β‐gal staining solution (1 mg/mL X‐gal, 40 mM citric acid/sodium phosphate buffer, pH 6.0, 5 mM potassium ferrocyanide, 5 mM potassium ferricyanide, 150 mM NaCl, and 2 mM MgCl_2_). After staining, sections were rinsed with PBS and counterstained with Nuclear Fast Red. SA‐β‐gal–positive areas (blue staining) were visualized under a bright‐field microscope and quantified using ImageJ software based on the percentage of positive area within five randomly selected cortical fields.

### 2.12 Generation of Primary Proximal Tubule‐Enriched Cells for Chromatin Immunoprecipitation Assay

Proximal tubule‐enriched cell populations were generated as previously described [44, 49, 50]. Briefly, kidney cortical tissue was finely minced and digested in RPMI 1640 (30264‐56; Nacalai Tesque) containing 1 mg/mL collagenase IV (034‐22363; Fujifilm Wako), 1 mg/mL dispase (17105‐041; Thermo Fisher Scientific), and 50 μg/mL DNase (90083; Thermo Fisher Scientific) for 45 min at 37°C with gentle shaking. The cell suspension was strained with a 70‐μm filter, washed with RPMI, collected by centrifugation three times, and cultured in RPMI 1640 with 5% FBS, 1 mM glucose, and penicillin/streptomycin. After a 24 h incubation under hypoxic conditions (1% O_2_) in a Modular Incubator Chamber (MIC 101) (Billups‐Rothenberg, San Diego, CA, USA), with or without the addition of 6.4 mM MgSO_4_, cells were collected for subsequent analyses.

### 2.13 Chromatin Immunoprecipitation Assay

The chromatin immunoprecipitation (ChIP) assay was performed as previously described [13, 34, 43]. Immunoprecipitation of chromatin was performed using a Magna ChIP A/G Chromatin Immunoprecipitation kit (16‐663; Merck Millipore, Billerica, MA, USA) with an anti‐H3K4me3 antibody (ab8580; Abcam) and normal IgG, incubated overnight at 4°C. DNA fragments were then purified using the QIAquick PCR Purification Kit (Qiagen, Valencia, CA, USA). Analysis of the p16^INK4a^ promoter region was performed using the CFX96 Real‐Time System (Bio‐Rad), with SYBR Green Premix Ex Taq (Takara Bio, Kusatsu, Japan) and the following pair of mouse *p16* ^*INK4a*^ primers, located at −1811 and −1706 bp from the transcription start site, respectively: forward, 5′‐GATGGAGCCCGGACTACAGAAG‐3′; and reverse, 5′‐CTGTTTCAACGCCCAGCTCTC‐3′.

### 2.14 Hypoxic Stimulation of Primary Renal Proximal Tubular Cells

Primary cultures of mouse renal proximal tubular cells were exposed to hypoxic conditions (1% O_2_) using a hypoxia chamber for 24 or 72 h, with or without MgSO_4_ treatment (6.4 mM). For qPCR analysis and ChIP assays, cells were collected after 24 h hypoxic stimulation. For western blot analysis of p16^INK4a^ protein expression, cells were exposed to hypoxia for 72 h before sample collection.

### 2.15 In Vitro Irradiation‐Induced Senescence Model

Primary mouse renal proximal tubular cells were exposed to a single dose of 8 Gy irradiation with or without MgSO_4_ treatment. SA‐β‐gal staining was performed at Day 7 after irradiation to evaluate senescence‐associated phenotypes.

### 2.16 Statistical Analyses

Results are presented as means ± SEM. Between‐group comparisons were performed using one‐way ANOVA followed by Tukey's post hoc test, as indicated in the figure legends. Survival analysis was conducted using the Kaplan–Meier method with a log‐rank test. A *p* value < 0.05 was considered statistically significant. Analyses were performed using GraphPad Prism software (version 10.4.2; GraphPad, San Diego, CA, USA).

## 3 Results

### 3.1 Mg^2+^ Reduces Renal Radiation Damage In Vitro and In Vivo

**FIGURE 1:**
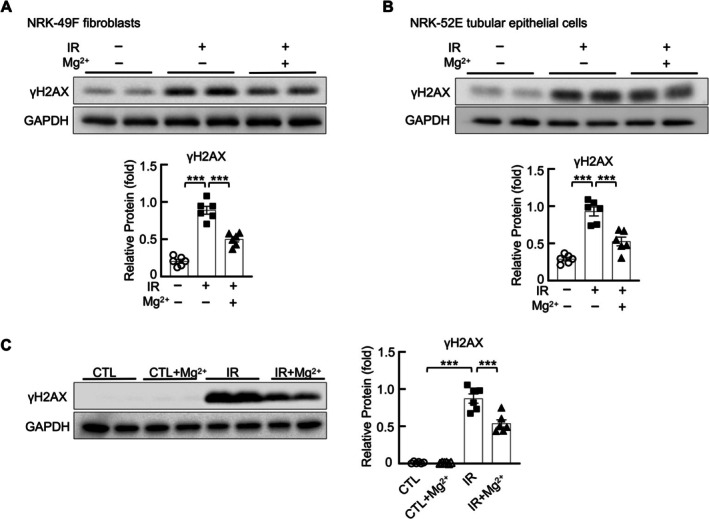
Effects of Mg^2+^ on radiation‐induced DNA damage in vitro and in vivo. (A, B) Representative western blots showing γH2AX expression 30 min after radiation‐induced injury (1 Gy) in NRK‐49F fibroblasts (A) and NRK‐52E tubular epithelial cells (B), with or without 6.4 mM MgSO_4_ treatment. Quantitative analyses are shown below each panel. (C) Representative western blot showing γH2AX expression in the kidneys of mice at 30 min post radiation‐induced injury (8 Gy), with or without pretreatment with Mg^2+^. Quantitative analysis is shown on the right side of the panel. Protein levels were normalized to GAPDH. *n* = 6 per group. ***p < 0.001, analyzed by one‐way ANOVA followed by Tukey's post hoc test. CTL, control; IR, irradiation.

Several studies have demonstrated that Mg^2+^ diminishes DNA damage resulting from chromatin aggregation [19, 22, 24]. First, to verify this protective effect of Mg^2+^ against DNA damage, we exposed mice to a lethal (8 Gy) dose of radiation [51] and evaluated their survival rate at 180 days, with or without administration of 600 mg/kg MgSO_4_ 30 min prior to the irradiation. Surprisingly, Kaplan–Meier curves showed a dramatically increased survival rate in the Mg^2+^ group compared with that in the control group (Figure S1). In the control group, mortality was observed from Day 10 postirradiation; by Day 20, 88.9% of the mice had died, with only 5.6% surviving to Day 180. In the Mg^2+^‐treated group, mortality was similarly observed from Day 10 postirradiation; however, only 27.8% of the mice had died by Day 20, and no further deaths were observed throughout the 180‐day study period, yielding a 72% survival rate (Figure S1). To confirm the protective effect of Mg^2+^ pretreatment on radiation‐induced DNA damage to the kidneys, we compared the levels of phosphorylated H2AX (γH2AX), a marker of the DNA damage response to double‐strand breaks, in irradiated NRK‐49F and NRK‐52E cells. The elevation in protein levels of γH2AX following exposure to 1 Gy of radiation [41] was mitigated by supplementing the culture medium of NRK‐49F and NRK‐52E cells with Mg^2+^ prior to the irradiation (Figure 1A,B). To investigate whether Mg^2+^ administration could also confer protection against radiation‐induced kidney injury in vivo, we exposed mouse kidneys to 8 Gy of radiation. The kidney expression of γH2AX protein was increased following irradiation, but was notably attenuated in the group treated with Mg^2+^ compared to irradiation group (Figure 1C). These data suggest that Mg^2+^ treatment mitigates irradiation‐induced DNA damage in mice. To evaluate systemic Mg^2+^ exposure under the experimental dosing conditions used in this study, serum Mg^2+^ concentrations were additionally measured after MgSO_4_ administration. Serum Mg^2+^ levels showed a transient elevation and declined toward baseline within several hours (Figure S2).

### 3.2 Mg^2+^ Suppresses IRI‐Induced Renal Morphological Changes and Fibrosis in Mice

**FIGURE 2:**
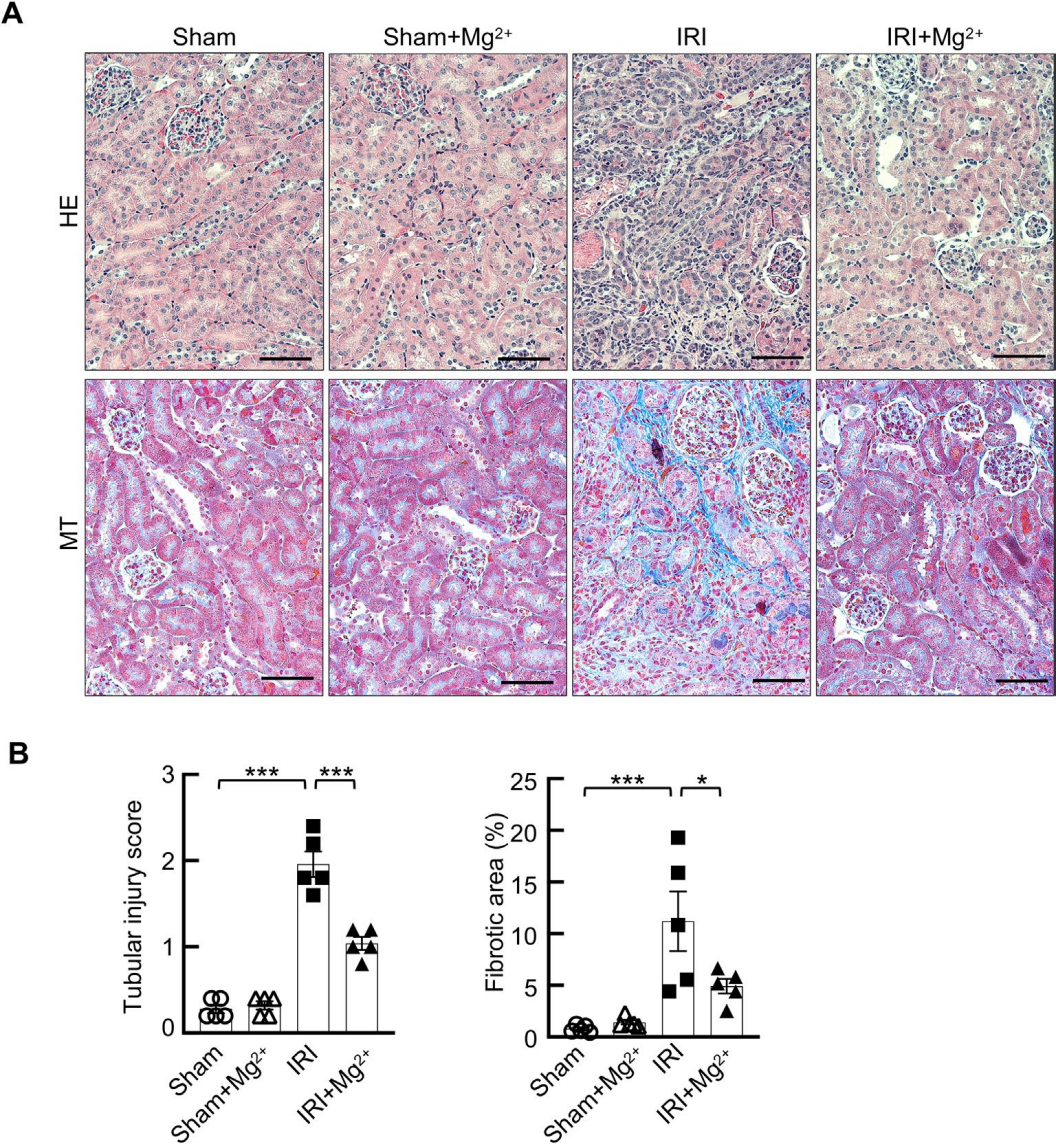
Effect of Mg^2+^ on the mouse kidney at Day 7 post‐IRI. (A) Representative hematoxylin–eosin (HE) and Masson's trichrome (MT) staining images of renal tissues in the Sham, Sham + Mg^2+^, IRI Day 7 (IRI), and IRI Day 7 + Mg^2+^ (IRI + Mg^2+^) treatment groups. Scale bars: 100 μm. (B) Quantitative analysis of staining results showing the tubular injury score and fibrotic area. *n* = 5 per group. *p < 0.05 and ***p < 0.001, analyzed by one‐way ANOVA followed by Tukey's post hoc test. HE, hematoxylin–eosin staining; IRI, ischemia–reperfusion injury; MT, Masson's trichrome.

DNA damage contributes to kidney injury and is often a precursor to CKD [52], leading us to hypothesize that Mg^2+^ might exert a protective effect against renal injury in the renal IRI mouse model [46, 53]. HE staining and Masson's trichrome (MT) staining of kidney tissues showed that IRI induced tubular damage and increased interstitial fibrosis in the kidney (Figure 2A,B). However, Mg^2+^ treatment significantly reduced both tubular damage and interstitial fibrosis compared to the IRI mice (Figure 2A,B). These data suggest that Mg^2+^ confers protection against IRI‐induced renal injury and fibrosis in mice.

### 3.3 Mg^2+^ Reduces DNA Damage‐ and DNA Repair‐Related Proteins Following IRI‐Induced Renal Injury in Mice

**FIGURE 3:**
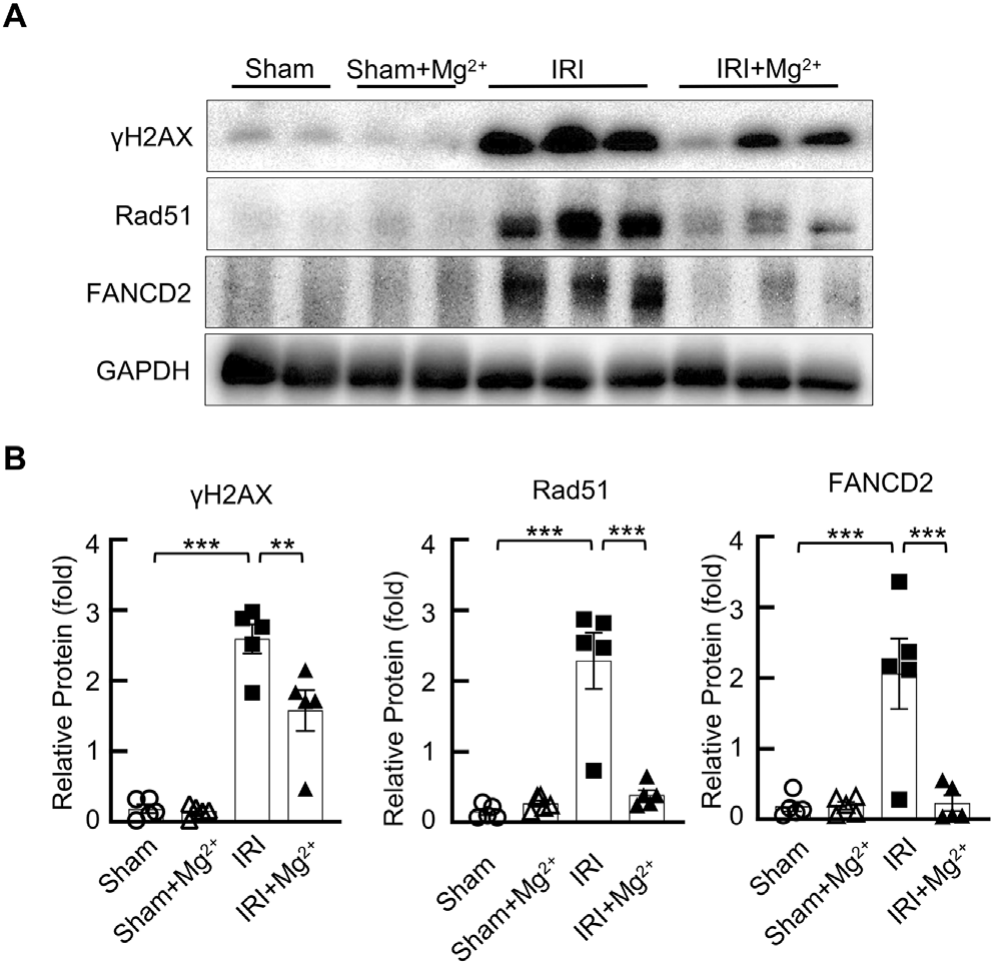
Effect of Mg^2+^ on DNA damage‐ and DNA repair‐related proteins in the mouse kidney at Day 7 post‐IRI. (A) Representative western blot showing the expression levels of γH2AX and the DNA repair‐related proteins Rad51 and FANCD2 in the kidneys from Sham, Sham + Mg^2+^, IRI Day 7 (IRI), and IRI Day 7 + Mg^2+^ (IRI + Mg^2+^) treatment groups. (B) Quantitative analysis of kidney expression of γH2AX, Rad51, and FANCD2 in the four groups. Protein levels were normalized to GAPDH. *n* = 5 per group. **p < 0.01 and ***p < 0.001, analyzed by one‐way ANOVA followed by Tukey's post hoc test. IRI, ischemia–reperfusion injury.

To ascertain whether the protective effect of Mg^2+^ against IRI injury is exerted through the reduction in DNA damage, we assessed the levels of DNA damage‐related proteins in mouse kidneys. Western blotting showed that the levels of γH2AX, radiation‐sensitive 51 (Rad51), and Fanconi anemia complementation group D2 (FANCD2), key proteins involved in DNA damage and repair pathways, were elevated in the kidneys of IRI group mice, but not sham group mice, reflecting increased DNA damage and repair demand. However, these elevations were attenuated by treatment with Mg^2+^, suggesting that Mg^2+^ reduced the extent of DNA damage and consequently decreased the activation of DNA repair pathways (Figure 3A,B). These data suggest that the protective effect of Mg^2+^ against renal injury is associated with decreased DNA damage.

### 3.4 Mg^2+^ Suppresses Renal Senescence‐Associated Signaling Following IRI‐Induced Renal Injury in Mice

**FIGURE 4:**
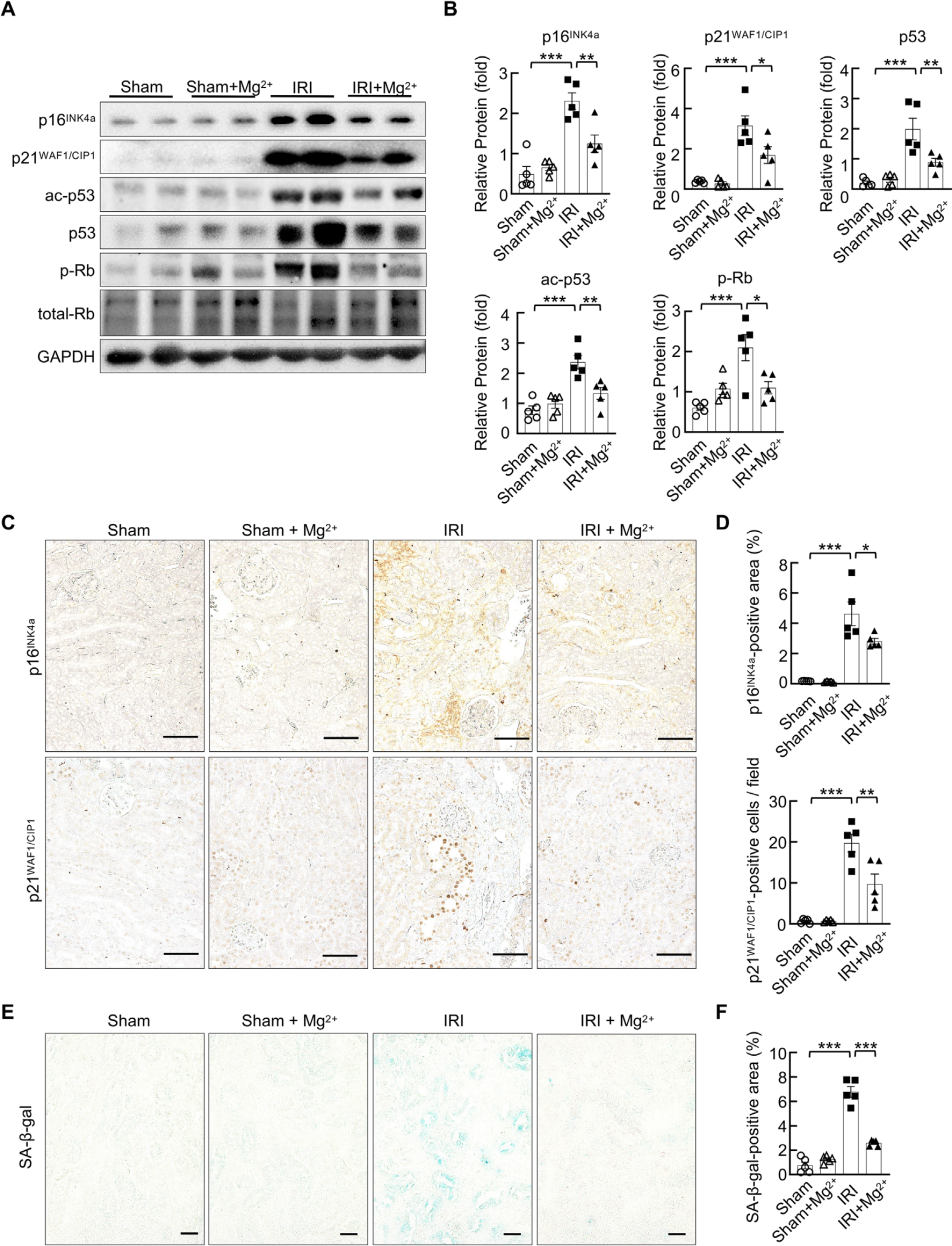
Effect of Mg^2+^ on senescence‐associated signaling in the mouse kidney at Day 7 post‐IRI. (A) Representative western blot showing the expression levels of p16^INK4a^, p21^WAF1/CIP1^, p53, acetylated p53 (ac‐p53), phosphorylated Rb (p‐Rb), and total‐Rb in the kidneys from the Sham, Sham + Mg^2+^, IRI Day 7 (IRI), and IRI Day 7 + Mg^2+^ (IRI + Mg^2+^) treatment groups. (B) Quantitative analysis of kidney levels of p16^INK4a^, p21^WAF1/CIP1^, p53, and ac‐p53 normalized to GAPDH, and p‐Rb normalized to total‐Rb, in the four groups. *p < 0.05, **p < 0.01, and ***p < 0.001, analyzed by one‐way ANOVA followed by Tukey's post hoc test. (C) Representative immunohistochemical staining images showing the expression of p16^INK4a^ and p21^WAF1/CIP1^ in renal tissues from the four groups. (D) Quantitative analysis of the p16^INK4a^‐positive area and p21^WAF1/CIP1^‐positive cell counts in kidneys from the four groups. *p < 0.05, **p < 0.01, and ***p < 0.001, analyzed by one‐way ANOVA followed by Tukey's post hoc test. (E) Representative images showing the senescence‐associated β‐galactosidase (SA‐β‐gal) staining in renal tissues from the four groups. (F) Quantitative analysis of the SA‐β‐gal–positive area in kidneys from the four groups. *n* = 5 per group. ***p < 0.001, analyzed by one‐way ANOVA followed by Tukey's post hoc test. Scale bars: 100 μm. IRI, ischemia–reperfusion injury.

Accumulation of unrepaired DNA damage within cells can serve as a driver of cellular senescence, contributing to stress‐induced senescence‐associated phenotypes [10]. Renal senescence arises from aging as well as various stimuli, including IRI, contributing to kidney fibrosis and inflammation [13, 14]. To evaluate the effect of Mg^2+^ on renal senescence, we used western blotting to examine the kidney levels of the cellular senescence markers p16^INK4a^, p21^WAF1/CIP1^, and p53, as well as acetylated p53 (ac‐p53), phosphorylated Rb (p‐Rb), and total Rb, following IRI. Compared with the sham surgery group mice, the IRI group mice showed increased levels of p16^INK4a^, p21^WAF1/CIP1^, p53, ac‐p53, and p‐Rb, whereas total Rb expression showed no marked difference among the groups. Administration of Mg^2+^ in the IRI group mice significantly suppressed the kidney levels of p16^INK4a^, p21^WAF1/CIP1^, p53, ac‐p53, and p‐Rb compared with IRI group mice (Figure 4A,B). These findings are consistent with activation of senescence‐associated pathways following IRI, which was attenuated by Mg^2+^ treatment. Immunohistochemical analysis further showed that the positive area of p16^INK4a^ staining and the number of p21^WAF1/CIP1^‐positive cells in the kidneys were increased following IRI compared with that following sham surgery. However, Mg^2+^ treatment in IRI mice significantly reduced the IRI‐induced p16^INK4a^ positive area and the number of p21^WAF1/CIP1^‐positive cells (Figure 4C,D). Furthermore, SA‐β‐gal staining revealed a marked increase in SA‐β‐gal–positive tubular cells in the IRI group compared with the sham group, whereas Mg^2+^ treatment significantly attenuated this increase (Figure 4E,F). These results suggest that treatment with Mg^2+^ attenuates the renal cellular senescence associated with intracellular accumulation of DNA damage in IRI mice.

### 3.5 Mg^2+^ Suppresses Senescence‐Associated Secretory Phenotype (SASP)‐Related Inflammation and Attenuates cGAS‐STING Pathway Activation Following IRI‐Induced Renal Injury in Mice

**FIGURE 5:**
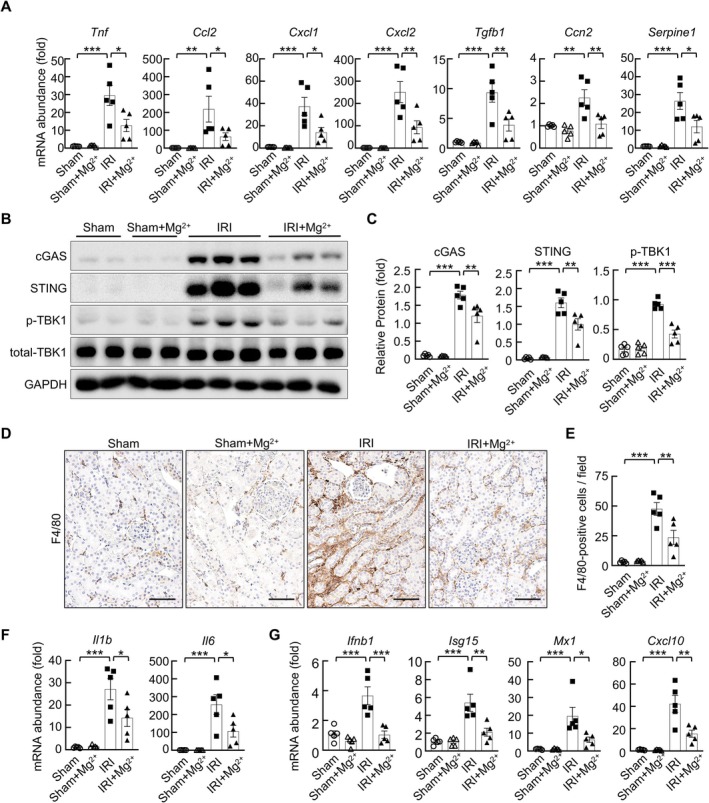
Effect of Mg^2+^ on SASP‐related gene expression, cGAS‐STING pathway activation, and inflammatory responses in mouse kidney at Day 7 post‐IRI. (A) qPCR analysis of the mRNA levels of *Tnf*, *Ccl2*, *Cxcl1*, *Cxcl2*, *Tgfb1*, *Ccn2*, and *Serpine1* in the kidneys from the Sham, Sham + Mg^2+^, IRI Day 7 (IRI), and IRI Day 7 + Mg^2+^ (IRI + Mg^2+^) treatment groups. Gene expression was normalized to the internal control *Gapdh*. *p < 0.05, **p < 0.01, and ***p < 0.001, analyzed by one‐way ANOVA followed by Tukey's post hoc test. (B) Representative western blot showing the expression levels of cGAS, STING, phosphorylated TBK1 (p‐TBK1), and total TBK1 in the kidneys from the four groups. (C) Quantitative analysis of the kidney levels of cGAS, and STING normalized to GAPDH, and p‐TBK1 normalized to total‐TBK1, in the four groups. **p < 0.01 and ***p < 0.001, analyzed by one‐way ANOVA followed by Tukey's post hoc test. (D) Representative immunohistochemical staining images showing the expression of F4/80 in renal tissues from the four groups. (E) Quantitative analysis of F4/80‐positive cells in kidneys from the four groups. **p < 0.01 and ***p < 0.001, analyzed by one‐way ANOVA followed by Tukey's post hoc test. (F) qPCR analysis of mRNA levels of *Il1b* and *Il6* in the kidneys from the four groups. Gene expression was normalized to the internal control *Gapdh*. *p < 0.05 and ***p < 0.001, analyzed by one‐way ANOVA followed by Tukey's post hoc test. (G) qPCR analysis of the mRNA levels of *Ifnb1*, *Isg15*, *Mx1*, and *Cxcl10* in kidneys from the four groups. Gene expression was normalized to *Gapdh*. *n* = 5 per group. *p < 0.05, **p < 0.01, and ***p < 0.001, analyzed by one‐way ANOVA followed by Tukey's post hoc test. Scale bars, 100 μm. IRI, ischemia–reperfusion injury.

Accumulation of DNA damage from unrepaired severe DNA damage or cellular senescence causes DNA fragments to leak into the cytoplasm, activating the cGAS‐STING pathway [16]. Recent investigations have elucidated the pivotal role of this pathway in promoting renal fibrosis through the induction of an inflammatory cascade [18, 54]. In addition, representative SASP‐related inflammatory and profibrotic genes were markedly upregulated following IRI and attenuated by Mg^2+^ treatment (Figure 5A). To assess the effect of Mg^2+^ on the cGAS‐STING pathway after IRI, we examined the expression of cGAS and STING proteins in the kidneys. Western blot analysis showed that the kidney levels of cGAS and STING were increased in IRI mice compared with those in sham mice. However, these increases in cGAS and STING expression were suppressed by treatment with Mg^2+^ (Figure 5B,C). In addition, renal p‐TBK1 levels, a downstream marker associated with cGAS–STING pathway activation, were elevated following IRI and attenuated by Mg^2+^ treatment (Figure 5B,C). To evaluate the impact of Mg^2+^ on innate immunity in relation to activation of the cGAS‐STING pathway, we examined the activity of macrophages. As shown in Figure 5D, immunohistochemical staining revealed that treatment with Mg^2+^ suppressed the IRI‐mediated increase in the number of F4/80‐positive macrophages in the kidneys (Figure 5D,E). To confirm the effect of Mg^2+^ on cGAS‐STING pathway‐associated inflammation in the kidneys following IRI, we examined the expression of *Il1b* and *Il6* at the mRNA level. As expected, Mg^2+^ treatment lowered the IRI‐mediated elevations in *Il1b* and *Il6* expression in the kidneys (Figure 5F). These results suggest that Mg^2+^ attenuates inflammation in the kidneys in association with reduced activation of DNA damage‐associated innate immune signaling pathways, including the cGAS‐STING axis. Consistent with attenuation of downstream innate immune signaling, the IRI‐induced upregulation of interferon response‐related genes (*Ifnb1*, *Isg15*, *Mx1*, *Cxcl10*, *Irf7*, and *Ifit1*) was also suppressed by Mg^2+^ treatment (Figure 5G and Figure S3).

### 3.6 Mg^2+^ Suppresses IRI‐Induced Renal Fibrosis in Mice

**FIGURE 6:**
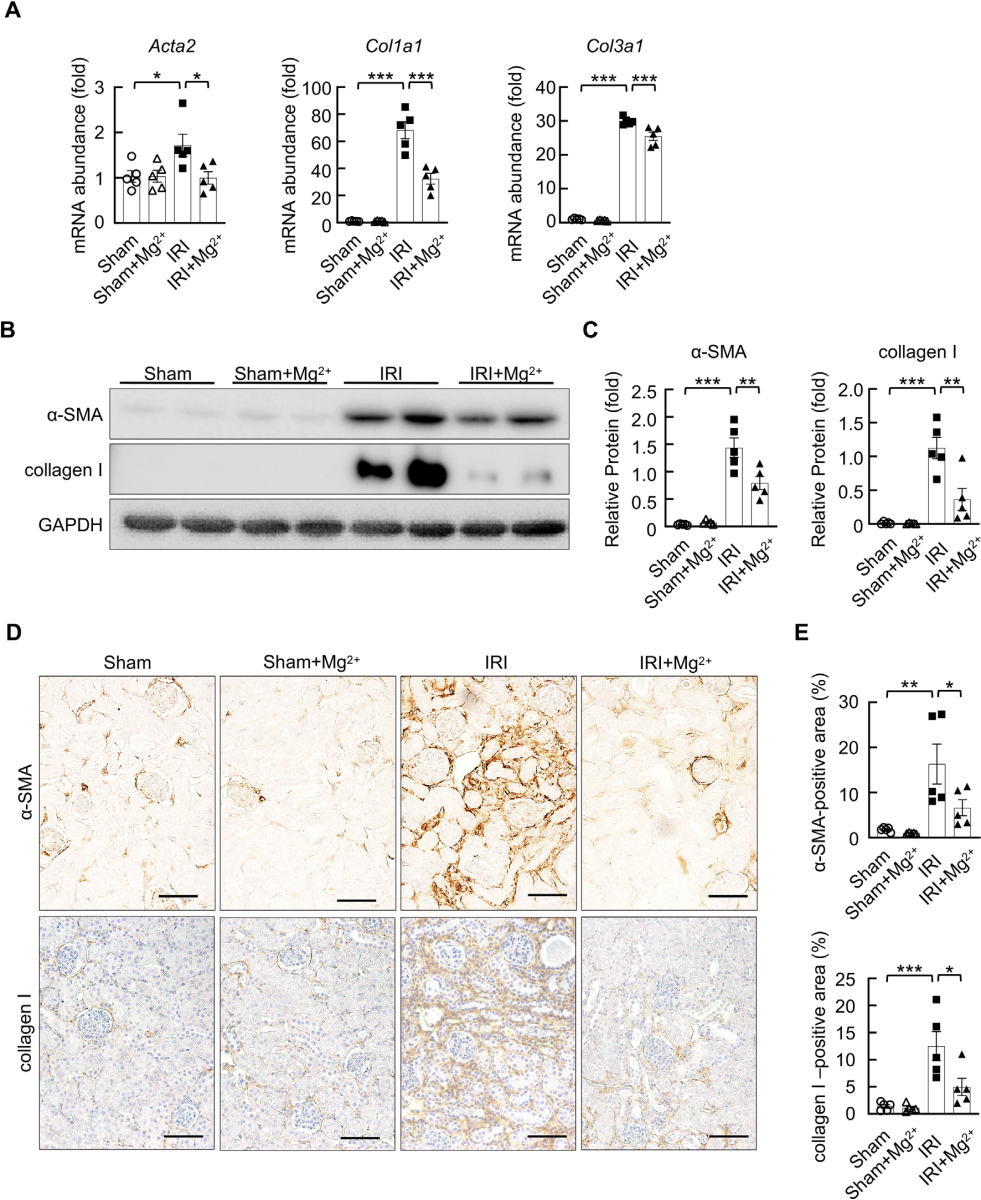
Effect of Mg^2+^ on renal fibrosis in the mouse kidney at Day 21 post‐IRI. (A) qPCR analysis of the mRNA levels of *Acta2*, *Col1a1*, and *Col3a1* in the Sham, Sham + Mg^2+^, IRI Day 21 (IRI), and IRI Day 21 + Mg^2+^ (IRI + Mg^2+^) treatment groups. Gene expression was normalized to the internal control *Gapdh*. *n* = 5 per group. *p < 0.05 and ***p < 0.001, analyzed by one‐way ANOVA followed by Tukey's post hoc test. (B) Representative western blot showing the expression levels of α‐SMA and collagen I in kidneys from the four groups. (C) Quantitative analysis of the kidney levels of α‐SMA and collagen I levels in the four groups, normalized to GAPDH. **p < 0.01 and ***p < 0.001, analyzed by one‐way ANOVA followed by Tukey's post hoc test. (D) Representative immunohistochemical staining images showing the expression of α‐SMA and collagen I in renal tissues from the four groups. (E) Quantitative analysis of the α‐SMA‐positive and collagen I‐positive areas in renal tissues from the four groups. *n* = 5 per group. *p < 0.05, **p < 0.01, and ***p < 0.001, analyzed by one‐way ANOVA followed by Tukey's post hoc test. Scale bar, 100 μm. IRI, ischemia–reperfusion injury.

To confirm the effects of Mg^2+^ administration on renal fibrosis, we examined the expression of profibrotic markers in kidney tissue. qPCR analysis at 3 weeks post‐surgery revealed significant upregulation of three profibrotic genes, namely *Acta2, Col1a1*, and *Col3a1*, in the IRI group compared with that in the sham group. By contrast, IRI mice treated with Mg^2+^ showed a significant decrease in the mRNA levels of *Acta2, Col1a1*, and *Col3a1* (Figure 6A). Furthermore, western blotting and immunohistochemical staining analyses demonstrated that Mg^2+^ treatment reduced the IRI‐mediated elevations in α‐smooth muscle actin (α‐SMA) and collagen I proteins (Figure 6B–E).

### 3.7 Mg^2+^ Suppresses p16^INK4a^ Expression and Senescence‐Associated Responses in Association With Altered Epigenetic Regulation in Experimental Models of Renal Injury

**FIGURE 7:**
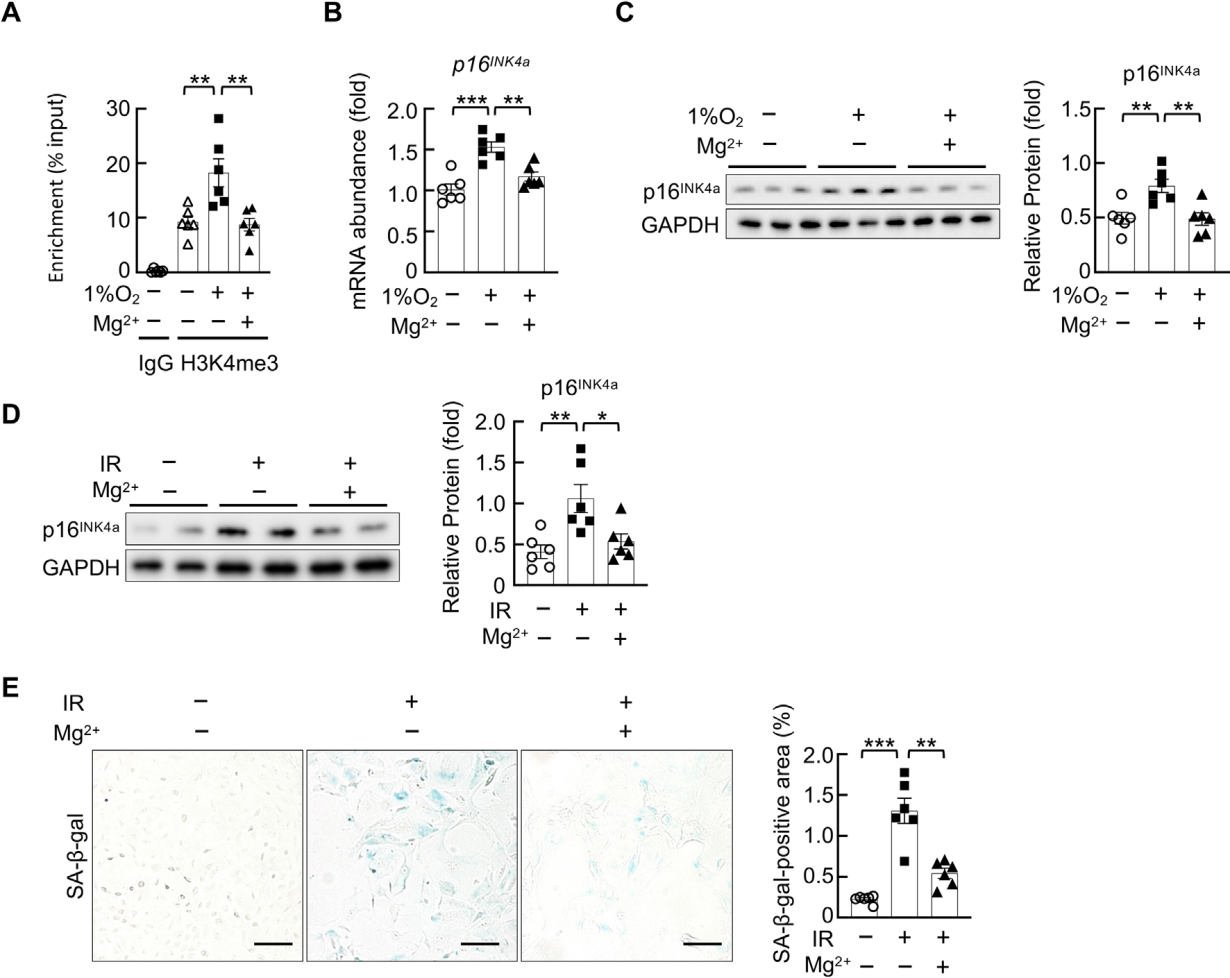
Effect of Mg^2+^ on p16^INK4a^ epigenetic regulation and senescence‐associated responses in experimental models of renal injury. (A) Relative fold enrichment of immunoprecipitated genomic fragments measured by qPCR in primary cultures of mouse renal proximal tubular cells exposed to hypoxia (1% O_2_), with or without Mg^2+^ treatment. Results were normalized to input DNA. *n* = 6 per group. **p < 0.01, analyzed by one‐way ANOVA followed by Tukey's post hoc test. (B) qPCR analysis of *p16* ^*INK4a*^ mRNA expression in primary cultures of mouse renal proximal tubular cells exposed to hypoxia (1% O_2_), with or without Mg^2+^ treatment under the same experimental conditions used for ChIP analysis. Gene expression was normalized to *Rps18*. *n* = 6 per group. **p < 0.01 and ***p < 0.001, analyzed by one‐way ANOVA followed by Tukey's post hoc test. (C) Representative western blot and quantitative analysis of p16^INK4a^ protein expression in primary cultures of mouse renal proximal tubular cells following prolonged hypoxic stimulation (72 h) with or without Mg^2+^ treatment. Protein levels were normalized to GAPDH. *n* = 6 per group. **p < 0.01, analyzed by one‐way ANOVA followed by Tukey's post hoc test. (D) Representative western blot and quantitative analysis of p16^INK4a^ protein expression in kidneys from control (CTL), irradiation (8 Gy; IR), and irradiation + Mg^2+^ treatment (IR + Mg^2+^) groups at Day 7 after irradiation. Protein levels were normalized to GAPDH. *n* = 6 per group. *p < 0.05 and **p < 0.01, analyzed by one‐way ANOVA followed by Tukey's post hoc test. (E) Representative senescence‐associated β‐galactosidase (SA‐β‐gal) staining images and quantitative analysis in primary mouse renal proximal tubular cells at Day 7 after 8 Gy irradiation, with or without Mg^2+^ treatment. *n* = 6 per group. **p < 0.01 and ***p < 0.001, analyzed by one‐way ANOVA followed by Tukey's post hoc test. Scale bars, 100 μm.

Mg^2+^ functions as a potential epigenetic regulator by altering chromatin structure [21, 35, 36]. We previously reported that epigenetic regulation of H3K4me3 suppresses mRNA expression of *p16* ^*INK4a*^ and attenuates both renal and peritoneal fibrosis [13, 34]. Here, the suppression of *p16* ^*INK4a*^ expression following Mg^2+^ administration (Figure 4) suggested a potential involvement of altered H3K4me3‐mediated epigenetic regulation. To determine whether this histone modification was associated with transcription of the *p16* ^*INK4a*^ gene under hypoxic conditions, we performed ChIP assays of primary cultures of mouse renal proximal tubular cells. This revealed that exposure of the cells to 1% O_2_ increased H3K4me3 enrichment at the *p16* ^*INK4a*^ promoter. However, treatment of the cells with Mg^2+^ during hypoxic induction significantly reduced H3K4me3 enrichment at this promoter (Figure 7A), confirming an association between Mg^2+^ treatment and reduced H3K4me3 enrichment at the *p16* ^*INK4a*^ promoter. To further strengthen the observed association between H3K4me3 modification and p16^INK4a^ expression, we evaluated *p16* ^*INK4a*^ mRNA expression under the same experimental conditions used for the ChIP assay. Hypoxic stimulation significantly increased *p16* ^*INK4a*^ mRNA expression, whereas Mg^2+^ treatment attenuated this increase (Figure 7B). Although p16^INK4a^ protein expression was not sufficiently induced after 24 h hypoxic stimulation under identical conditions (Figure S4), prolonged hypoxic stimulation for 72 h induced a marked increase in p16^INK4a^ protein expression, which was attenuated by Mg^2+^ treatment (Figure 7C). To further confirm the association between DNA damage and senescence in an independent experimental setting, we examined p16^INK4a^ expression in the irradiation (8 Gy) Day 7 model. Irradiation significantly increased renal p16^INK4a^ protein expression, whereas Mg^2+^ treatment suppressed this increase (Figure 7D). Furthermore, primary mouse renal proximal tubular cells exposed to 8 Gy irradiation exhibited increased SA‐β‐gal positivity at Day 7, which was attenuated by Mg^2+^ treatment (Figure 7E). These findings further support that Mg^2+^ suppresses senescence‐associated responses, along with altered epigenetic regulation and attenuated DNA damage‐associated cellular stress.

## 4 Discussion

Our study has revealed that Mg^2+^ plays a crucial role in attenuating renal fibrosis by reducing DNA damage and tissue injury while regulating the expression of the senescence marker p16^INK4a^ in the mouse kidney. Notably, Mg^2+^ exhibited a protective effect against lethal radiation‐induced organ damage in mice. It also alleviated radiation‐induced DNA damage both in vitro and in vivo, and suppressed IRI‐induced morphological changes, DNA damage, and cellular senescence in the mouse kidney. Additionally, Mg^2+^ reduced tissue inflammation and cGAS‐STING pathway activation, along with attenuation of post‐IRI renal fibrosis. Finally, we demonstrated that Mg^2+^ treatment decreased *p16* ^*INK4a*^ transcription and reduced H3K4me3 enrichment at the *p16* ^*INK4a*^ promoter, together with chromatin structural changes, in primary cultures of mouse renal proximal tubular cells. These findings suggest that Mg^2+^‐induced chromatin dynamics have potential as a therapeutic strategy to prevent renal senescence and fibrosis.

Previous studies have reported that Mg^2+^ reduces DNA damage by enhancing genomic stability through alterations in higher‐order chromatin structure [19, 24, 28], not only via DNA charge neutralization [55], but also through non‐electrostatic mechanisms such as the intracellular Mg^2+^–ATP balance [22]. Our findings corroborate the organ‐protective effects of Mg^2+^ against DNA damage [28]. Although we did not specifically investigate the cause of death in our experiments, total‐body irradiation (TBI) of mice at 8 Gy is generally reported to result in mortality due to a combination of factors, including bone marrow suppression‐associated hematopoietic acute radiation syndrome (ARS) and gastrointestinal ARS [56, 57]. According to Kumar et al. [57], the 30‐day survival rate following 8 Gy TBI in C57BL/6 mice is 13%. They further reported that administration of a novel thrombopoietin mimetic, JNJ‐26366821, an analog of drugs approved by the FDA for mitigating hematopoietic ARS, increased the 30‐day post‐TBI survival rate to 83%. In our study, the 27‐day survival rate in the non Mg^2+^ control group was 11.1%, which was similar to that reported by Kumar et al.; however, one mouse later died, resulting in a 30‐day survival rate of 5.6%. Additionally, the 30‐day survival rate with JNJ‐26366821 was 83%, which is only slightly better than the 72% observed in our Mg^2+^‐treated group. Because Mg^2+^ is readily available as a laxative, it may offer a more convenient approach to improve survival rates following radiation exposure. Our findings suggest the potential for new clinical applications of magnesium dynamics, extending beyond renal protection against DNA damage to include defense against lethal radiation injury.

Our demonstration that Mg^2+^ administration can reduce renal DNA damage and inhibit IRI‐induced renal fibrosis is an important finding. Renal fibrosis has been associated with increased inflammation [5, 58], oxidative stress [5, 59], and apoptosis [5, 60]. Furthermore, multiple studies have documented the anti‐inflammatory [26], antioxidant [27, 28], and anti‐apoptotic [29] effects of Mg^2+^, suggesting its potential to attenuate renal fibrosis [61]. Although there are relatively few reports on Mg^2+^ administration improving renal fibrosis, it has indeed been shown to attenuate renal fibrosis induced by cyclosporine [62] and aldosterone [25]. A study by Pundir et al. also reported that Mg^2+^ ameliorated renal tissue damage in IRI‐induced acute kidney injury [63], consistent with our findings in the IRI‐induced renal fibrosis model. In cardiomyocytes, Mg^2+^ has also been shown to exert beneficial effects on fibrosis [64], suggesting that Mg^2+^ administration may represent a potential therapeutic approach for the treatment of fibrotic diseases in other organs.

In this study, Mg^2+^ administration suppressed the expression of p16^INK4a^, p21^WAF1/CIP1^, and p53, thereby attenuating renal fibrosis. Cellular senescence has been linked to fibrosis in various organs [65, 66]; however, its role in renal fibrosis appears to be complex [67]. While some studies suggest that senescent cells have beneficial effects in the early stages [68], their long‐term consequences may be detrimental [69, 70]. We previously reported that inhibition of the histone methyltransferase complex MLL1/WDR5 suppresses p16^INK4a^ expression and attenuates renal and peritoneal fibrosis [13, 34], consistent with the findings of the present study. There is accumulating evidence linking Mg^2+^ deficiency to aging and age‐related diseases [71, 72]. Interestingly, dietary Mg^2+^ supplementation in a mouse model of premature aging was even shown to enhance mitochondrial function and extend lifespan [73]. Thus, Mg^2+^—widely available as an over‐the‐counter supplement for various physiological functions—may influence senescence‐associated cellular phenotypes.

Severe unrepaired DNA damage or senescence‐related accumulation of DNA damage can cause fragmented DNA to leak into the cytoplasm, where it is recognized by cGAS, resulting in activation of the cGAS–STING pathway [74]. Furthermore, cGAS is essential for cellular senescence [17], and inflammation mediated by the STING pathway has been shown to contribute to renal fibrosis [18]. In the current study, Mg^2+^ administration suppresses the cGAS–STING pathway, along with attenuation of senescence and improvement of renal fibrosis. The involvement of the cGAS–STING pathway in acute kidney injury and renal fibrosis has been previously reported [54, 75], consistent with our findings. One possible interpretation of our data showing the Mg^2+^‐mediated suppression of this pathway is that Mg^2+^ reduces DNA damage, as evidenced by the decreased levels of the DNA damage marker γH2AX. This reduction may contribute to decreased cytoplasmic leakage of DNA fragments. Indeed, numerous studies have reported that Mg^2+^ alleviates DNA damage [28]. However, cGAS–STING signaling may also be activated independently of DNA damage, potentially through mechanisms such as mitochondrial dysfunction and associated cellular stress responses. Therefore, although our findings support an association between reduced DNA damage and attenuation of DNA damage‐associated innate immune signaling pathways, including the cGAS–STING axis, additional mechanisms may also contribute to regulation of this pathway.

Here, Mg^2+^ treatment was associated with reduced H3K4me3 enrichment at the *p16* ^*INK4a*^ promoter and decreased p16^INK4a^ expression, in association with epigenetic chromatin remodeling. Mg^2+^ stabilizes DNA conformation through electrostatic interactions, hydrogen bonding, and the Mg^2+^–ATP balance, thereby contributing to both the secondary and tertiary structures of DNA [19, 22, 24]. Importantly, Mg^2+^ may also function as an epigenetic regulator by modulating chromatin architecture [21, 35, 36, 76]. Supporting this concept, Takaya et al. [77] reported that offspring born to Mg^2+^‐deficient pregnant rats exhibited DNA hypermethylation of the hepatic 11β‐hydroxysteroid dehydrogenase type 2 (*Hsd11b2*) gene promoter, which is associated with gene silencing and metabolic alterations in neonates. Furthermore, according to Lin et al., nucleosomes adopt a more compact structure in the presence of Mg^2+^ and K^+^. While ionic conditions have a smaller influence than posttranslational modifications (PTMs) on structural changes induced by nucleosome modifications, they observed a clear impact of PTMs under these ionic conditions [78]. While most of the changes in histone modifications identified in the field of renal failure have been limited to specific gene loci [79], studies in cancer and epigenetics have also detected global changes in histone modifications [80]. Whether Mg^2+^ exerts its role as an epigenetic regulator through locus‐specific mechanisms or broader alterations in histone modifications is an important question for future investigation.

Although different experimental models were used in this study, these approaches provided complementary insights into the role of Mg^2+^ in DNA damage‐associated senescence and epigenetic regulation. In the hypoxia‐based in vitro model used for mechanistic epigenetic analyses, hypoxic stimulation increased *p16* ^*INK4a*^ mRNA expression, which was attenuated by Mg^2+^ treatment under the same conditions used for the ChIP assay. Furthermore, prolonged hypoxic stimulation induced p16^INK4a^ protein expression, which was likewise suppressed by Mg^2+^ treatment. These findings support the functional association between reduced H3K4me3 enrichment at the *p16* ^*INK4a*^ promoter and suppression of *p16* ^*INK4a*^ expression. In parallel, radiation‐based models demonstrated increased p16^INK4a^ expression and SA‐β‐gal activity following severe DNA damage, both of which were attenuated by Mg^2+^ treatment. Collectively, these findings consistently support the suppressive effects of Mg^2+^ on DNA damage‐associated senescence across multiple experimental settings. Importantly, the experimental models used in this study primarily reflect injury‐induced senescence following radiation injury or ischemia–reperfusion injury rather than physiological aging itself.

The MgSO_4_ dosing conditions used in this study exceeded physiological magnesium levels; however, serum Mg measurements demonstrated only a transient elevation after administration, with concentrations declining toward baseline within several hours (Figure S2). MgSO_4_ was administered slowly by intraperitoneal injection to minimize acute systemic effects, and no treatment‐related mortality was observed following MgSO_4_ administration under our experimental conditions. Nevertheless, the optimal dosing strategy and clinical translatability of Mg^2+^ administration remain to be established. In addition, we cannot exclude potential contributions from systemic hemodynamic, renal blood flow, or metabolic effects, which were not directly evaluated in the present study, and these factors could potentially influence fibrosis independently of direct Mg^2+^‐mediated cellular mechanisms, particularly in the unilateral IRI model. Further studies are warranted to determine clinically relevant dosing conditions and to clarify potential nonspecific systemic effects.

In summary, this study highlights the potential role of Mg^2+^ in injury‐induced cellular senescence, inflammation, and renal fibrosis, and underscores the importance of epigenetic modifications in IRI‐induced fibrogenesis. Our findings demonstrate that Mg^2+^ treatment is associated with reductions in DNA damage‐associated cellular stress, cellular senescence, and cGAS‐STING pathway activation, along with reduced renal fibrosis. Furthermore, our findings indicate that Mg^2+^ attenuates injury‐induced cellular senescence along with altered epigenetic regulation, suggesting the potential of Mg^2+^ administration as a therapeutic strategy for targeting inflammation and fibrosis in association with reduced renal DNA damage and altered epigenetic modulation. Although clinically relevant Mg^2+^ dosing conditions and therapeutic applicability require further investigation, elucidating these intricate interactions may deepen our understanding of the mechanisms underlying fibrosis and senescence‐associated renal pathophysiology.

## Author Contributions

M.M., S.D., T.M., and K.S. conceived and designed the research. M.M., T.I., M.Y., A.T., Y.O., N.I., A.O., and Y.M. performed the research and acquired the data. M.M., K.S., T.C., A.N., and T.M. analyzed and interpreted the data. M.M. wrote the first draft of the manuscript, and K.S., T.C., and T.M. edited the manuscript. All authors commented on previous versions of the manuscript and approved the final manuscript.

## Funding

The authors have nothing to report.

## Conflicts of Interest

The authors declare no conflicts of interest.

## Supporting information


**Figure S1:** Effects of Mg^2+^ on survival from lethal IR. Representative Kaplan–Meier survival curves showing the survival rates of lethally irradiated mice at 180 days, with Mg^2+^ administration or without (control; CTL), analyzed and compared using the log‐rank test. Note the significantly higher survival rate in the Mg^2+^‐treated group. *n* = 18 per group.
**Figure S2:** Time course of serum Mg^2+^ concentrations following MgSO_4_ administration. Serum Mg^2+^ concentrations were measured in DDW‐treated and MgSO_4_‐treated mice at baseline (0 h) and 1, 2, and 6 h after administration under the experimental dosing conditions used in this study. Hemolyzed samples were excluded from analysis.
**Figure S3:** Additional interferon response‐related genes following renal ischemia–reperfusion injury. Renal mRNA expression levels of *Irf7* and *Ifit1* in sham, sham + Mg^2+^, IRI, and IRI + Mg^2+^ groups. IRI‐induced increases in *Irf7* and *Ifit1* expression were attenuated by Mg^2+^ treatment. Gene expression was normalized to *Gapdh* and analyzed using the 2^−ΔΔCt^ method. *n* = 5 per group. Data are presented as mean ± SEM. Analyzed by one‐way ANOVA followed by Tukey's post hoc test.
**Figure S4:** p16^INK4a^ protein expression under the same experimental conditions used for ChIP analysis. Representative western blot and quantitative analysis of p16^INK4a^ protein expression in primary cultures of mouse renal proximal tubular cells exposed to hypoxia (1% O_2_) with or without Mg^2+^ treatment under the same experimental conditions used for ChIP analysis (24 h). p16^INK4a^ protein expression was not sufficiently induced at 24 h after hypoxic stimulation. Protein levels were normalized to GAPDH. *n* = 6 per group. Data are presented as mean ± SEM. Analyzed by one‐way ANOVA followed by Tukey's post hoc test.
**Figure S5:** Full blots for Figure 1A–C.
**Figure S6:** Full blots for Figure 3A.
**Figure S7:** Full blots for Figure 4A.
**Figure S8:** Full blots for Figure 5B.
**Figure S9:** Full blots for Figure 6B.
**Figure S10:** Full blots for Figure 7C,D.
**Figure S11:** Full blots for Figure S4.

**Table S1:** Primer sequences used for SYBR Green quantitative PCR analysis.

**Data S1:** Source data underlying the quantitative analyses presented in Figures 1–7 and Supporting Information Figures S1–S4.

## Data Availability

All data supporting the findings of this study are included in the article and its Supporting Information, including source data files.
