# Supplementary material for: Magnesium Attenuates Renal Senescence and Fibrosis With Reduced DNA Damage Response and H3K4me3 Enrichment at the p16INK4a Promoter
Source: FASEB J. 2026 Aug 14;40(16):e72192. doi: 10.1096/fj.202504665RR (PMC13475955; doi:10.1096/fj.202504665RR)
Supplement: Supplementary file 1 [file FSB2-40-e72192-s003.pdf]

SUPPLEMENTAL MATERIAL

Magnesium Attenuates Renal Senescence and Fibrosis with Reduced DNA Damage Response and H3K4me3 Enrichment at the $p16^{INK4a}$ Promoter

Makoto Matsubara[1], Kensuke Sasaki[1]*(ORCID ID: 0000-0001-5899-0885), Shigehiro Doi[1], Takeshi Ike[1], Maria Yoshida[1], Akira Takahashi[1], Yosuke Osaki[1], Naoki Ishiuchi[1], Aiko Okubo[1], Yujiro Maeoka[1], Takuto Chiba[2], Ayumu Nakashima[3], and Takao Masaki[1]*

1. Department of Nephrology, Hiroshima University Hospital, Hiroshima, Japan.
2. Division of Nephrology, Department of Pediatrics, University of Pittsburgh School of Medicine, Pittsburgh, Pennsylvania, USA.
3. Department of Nephrology, Graduate School of Medicine, University of Yamanashi, Yamanashi, Japan.

Supplementary Figure Legends

Supplementary Figure S1. Effects of Mg^{2+} on survival from lethal IR.
Representative Kaplan-Meier survival curves showing the survival rates of lethally irradiated mice at 180 days, with Mg^{2+} administration or without (control; CTL), analyzed and compared using the log-rank test. Note the significantly higher survival rate in the Mg^{2+}-treated group. n = 18 per group.

Supplementary Figure S2. Time course of serum Mg^{2+} concentrations following $MgSO_4$ administration.
Serum Mg^{2+} concentrations were measured in DDW-treated and $MgSO_4$-treated mice at baseline (0 h) and 1, 2, and 6 h after administration under the experimental dosing conditions used in this study. Hemolyzed samples were excluded from analysis.

Supplementary Figure S3. Additional interferon response-related genes following renal ischemia-reperfusion injury.
Renal mRNA expression levels of Irf7 and Ifit1 in sham, sham+Mg^{2+}, IRI, and IRI+Mg^{2+} groups. IRI-induced increases in Irf7 and Ifit1 expression were attenuated by Mg^{2+} treatment. Gene expression was normalized to *Gapdh* and analyzed using the $2^{-\Delta\Delta Ct}$ method. n = 5 per group. Data are presented as mean ± SEM. Analyzed by one-way ANOVA followed by Tukey's post-hoc test.

Supplementary Figure S4. $p16^{INK4a}$ protein expression under the same experimental conditions used for ChIP analysis.
Representative western blot and quantitative analysis of $p16^{INK4a}$ protein expression in primary cultures of mouse renal proximal tubular cells exposed to hypoxia (1% O_2) with or without Mg^{2+} treatment under the same experimental conditions used for ChIP analysis (24 h). $p16^{INK4a}$ protein expression was not sufficiently induced at 24 h after hypoxic stimulation. Protein levels were normalized to GAPDH. n = 6 per group. Data are presented as mean ± SEM. Analyzed by one-way ANOVA followed by Tukey's post-hoc test.

Supplementary Figure S5. Full Blots for Figure 1A-C
Supplementary Figure S6. Full Blots for Figure 3A
Supplementary Figure S7. Full Blots for Figure 4A
Supplementary Figure S8. Full Blots for Figure 5B
Supplementary Figure S9. Full Blots for Figure 6B
Supplementary Figure S10. Full Blots for Figure 7C, D
Supplementary Figure S11. Full Blots for Supplementary Figure S4

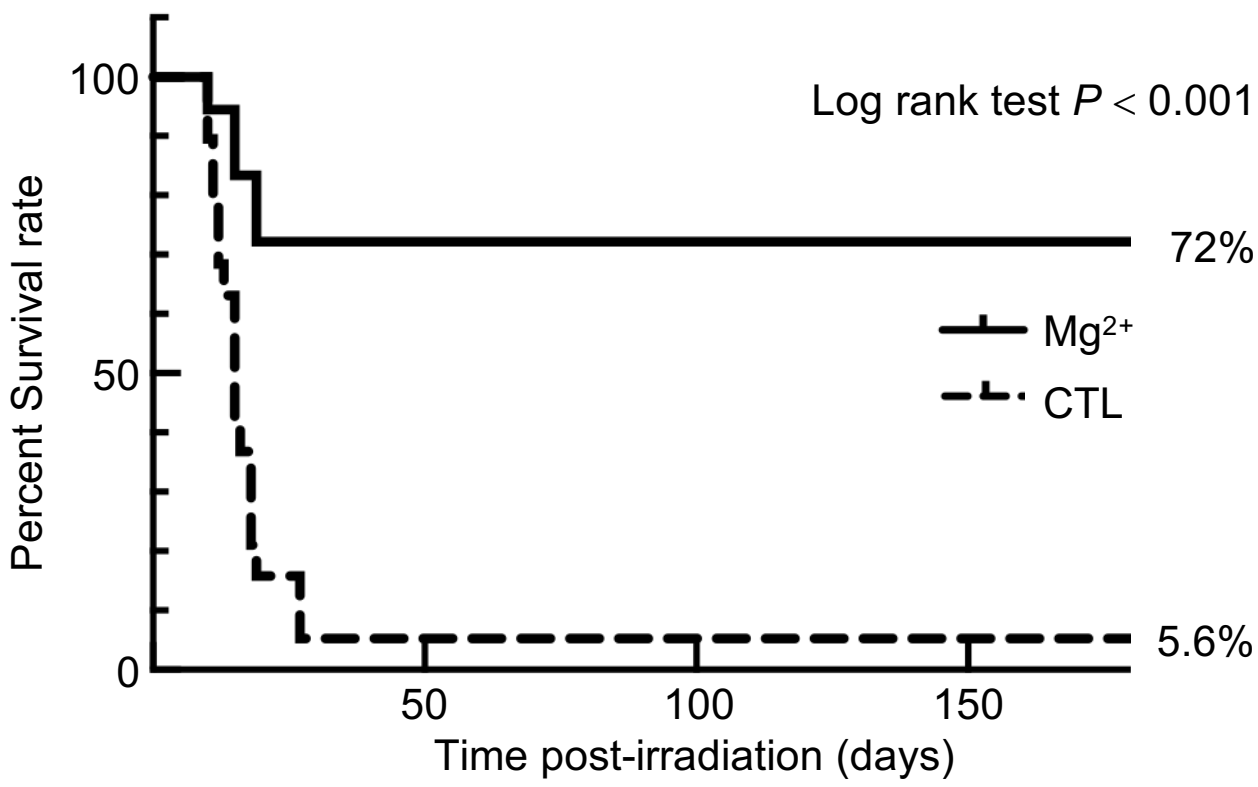

Supplementary Figure S1

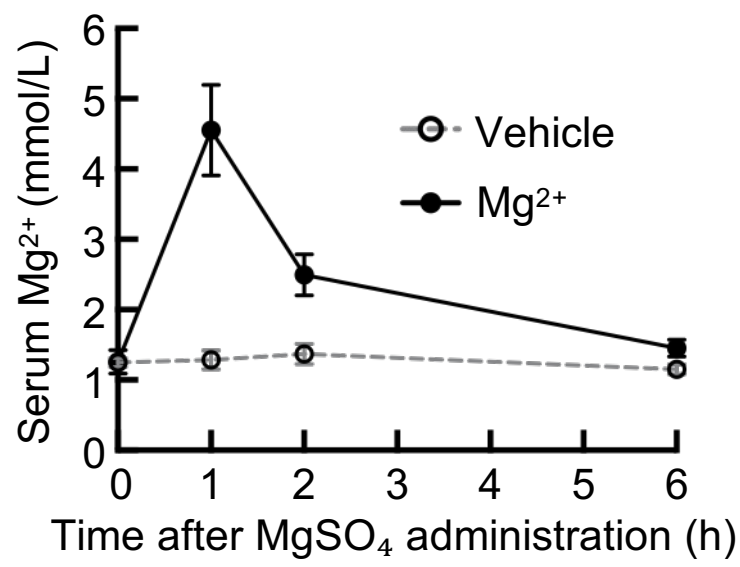

Supplementary Figure S2

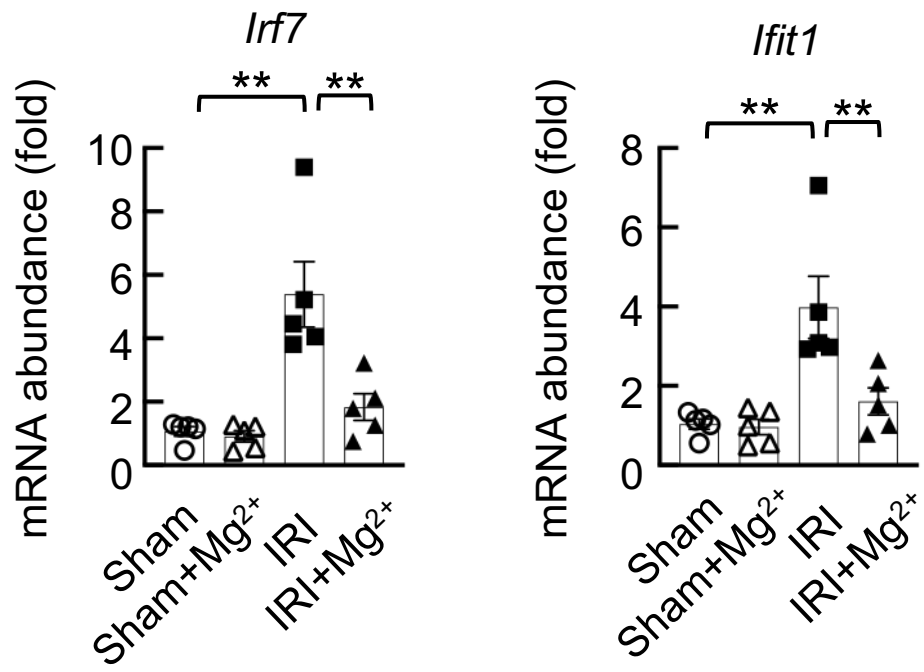

Supplementary Figure S3

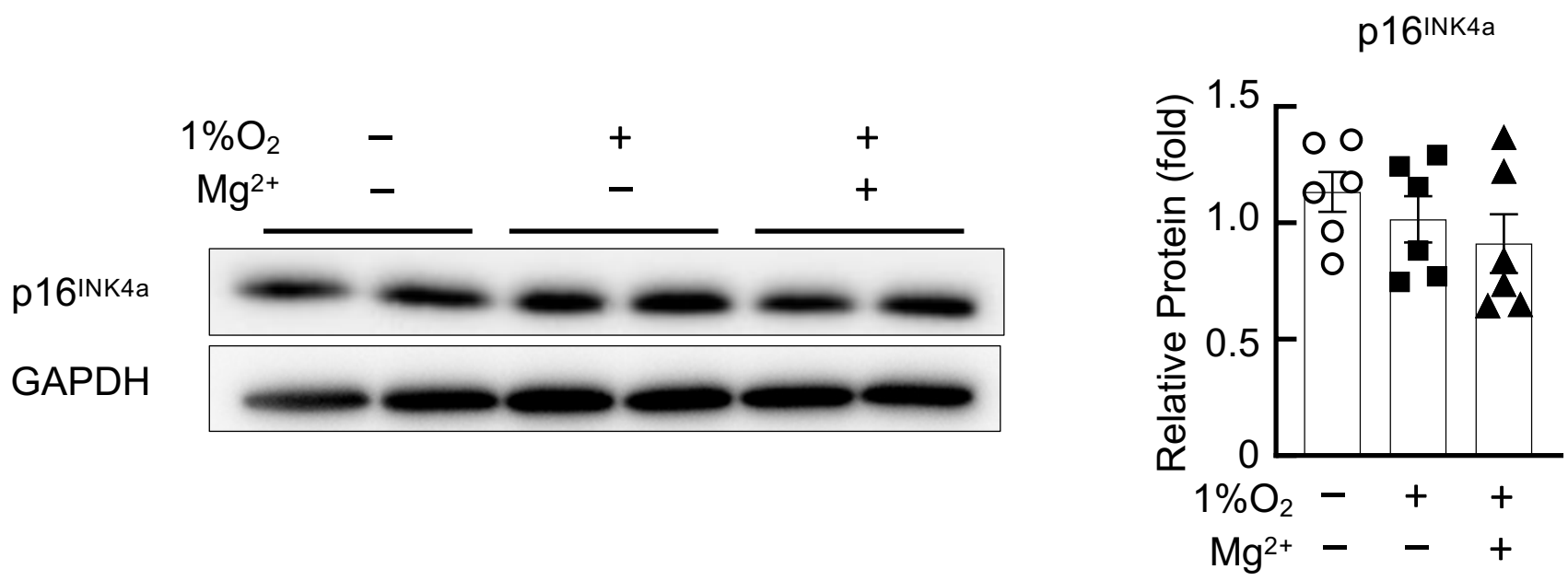

Supplementary Figure S4

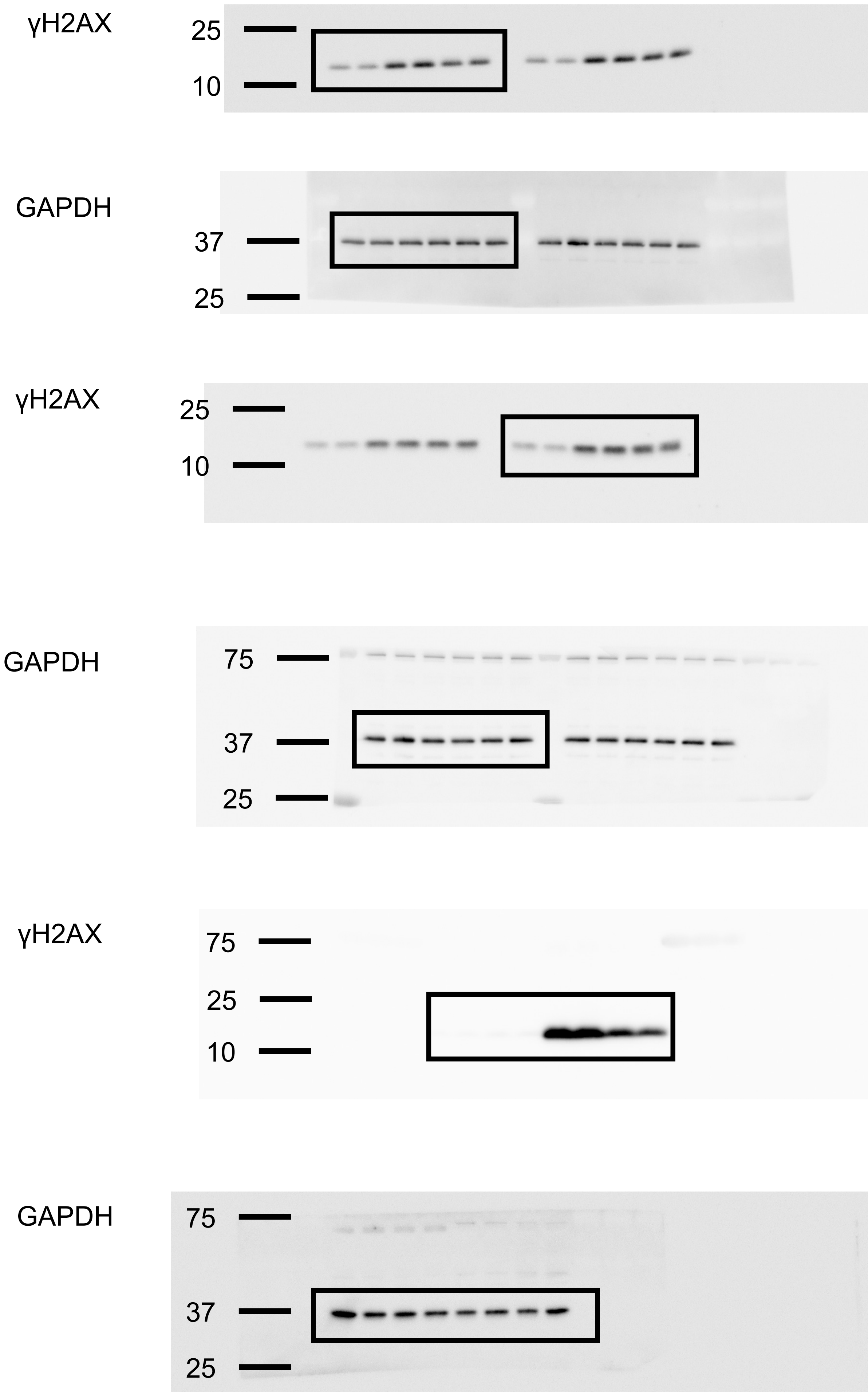

Supplementary Figure S5. Full Blots for Figure 1A-C

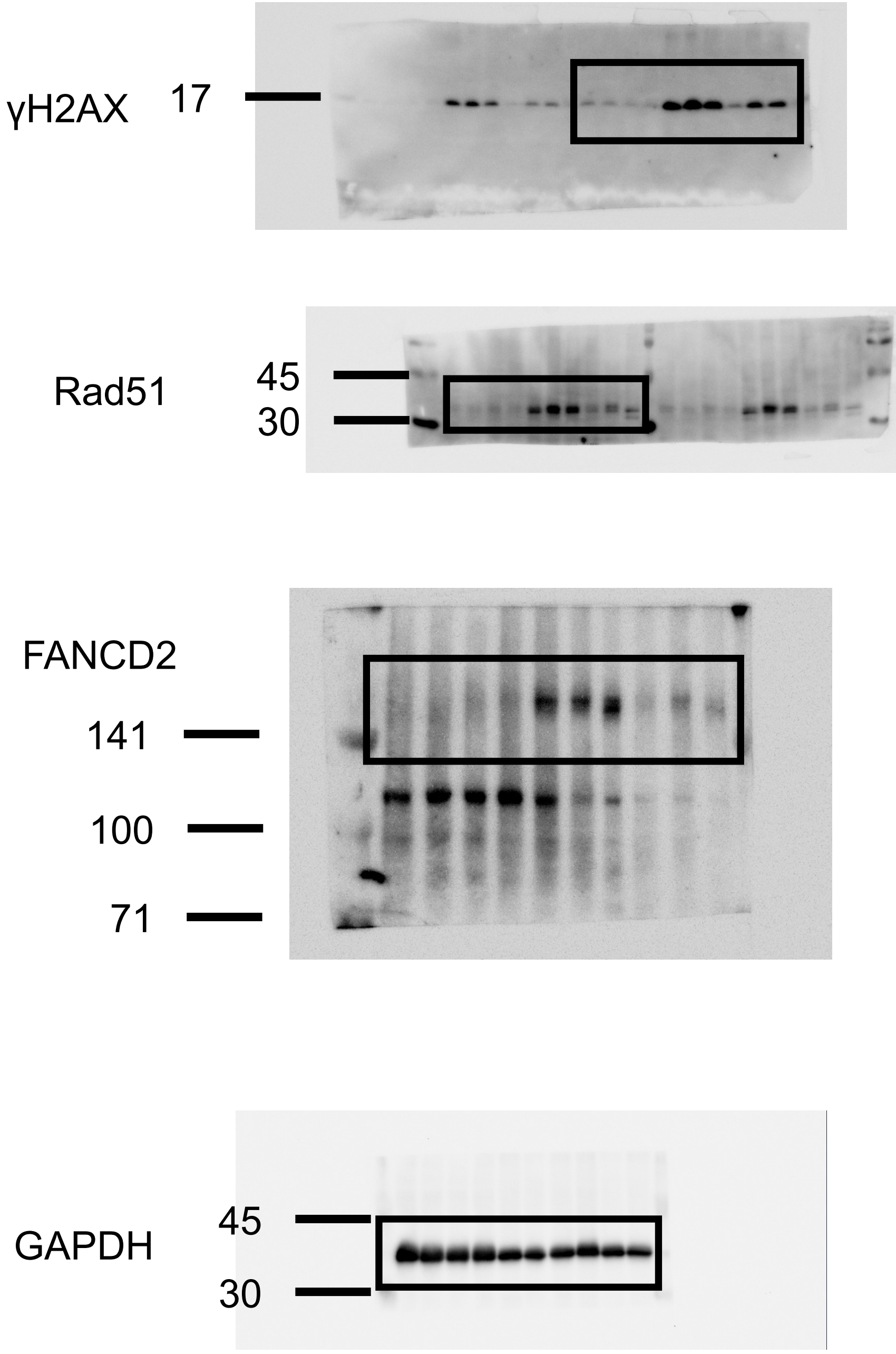

Supplementary Figure S6. Full Blots for Figure 3A

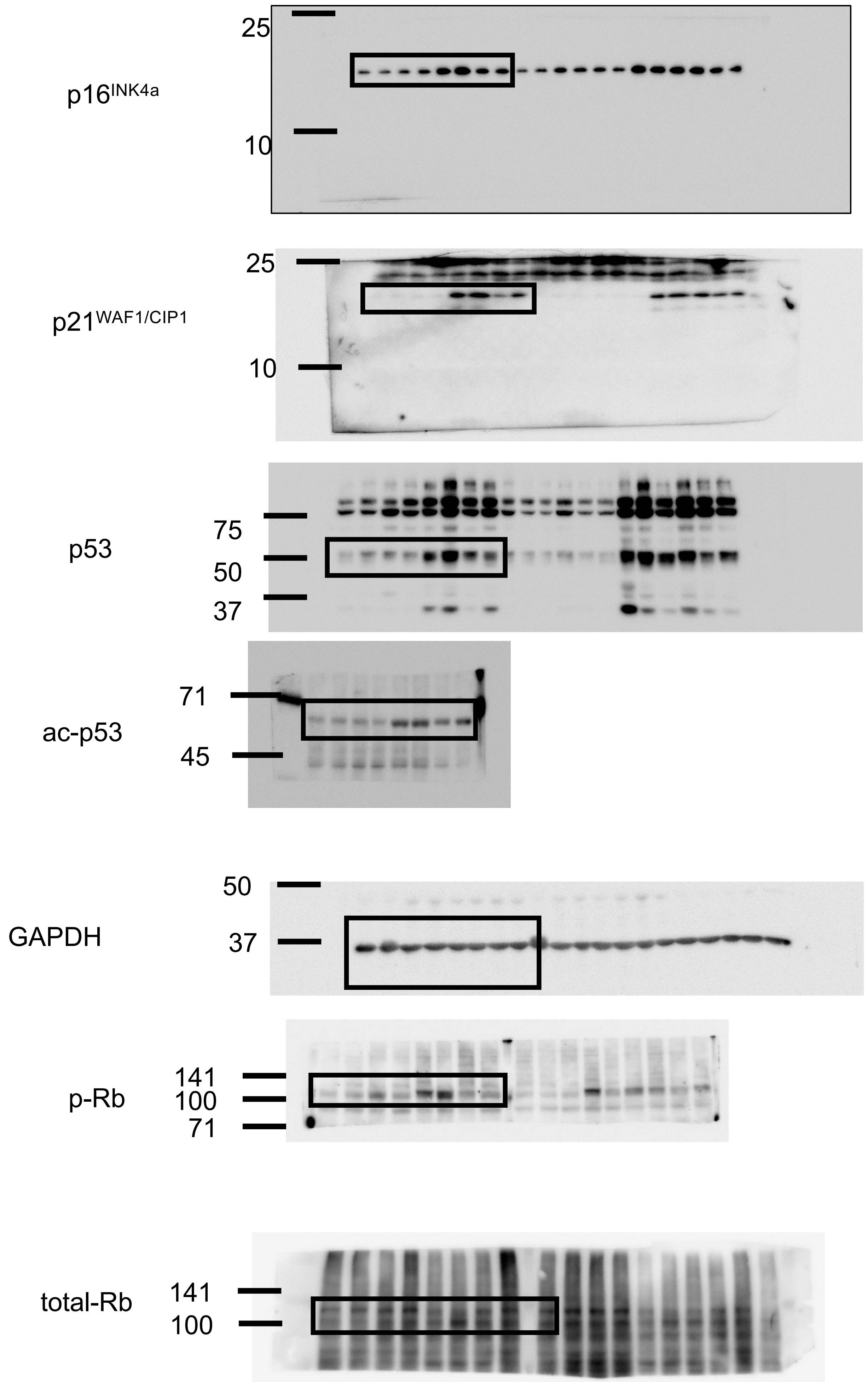

Supplementary Figure S7. Full Blots for Figure 4A

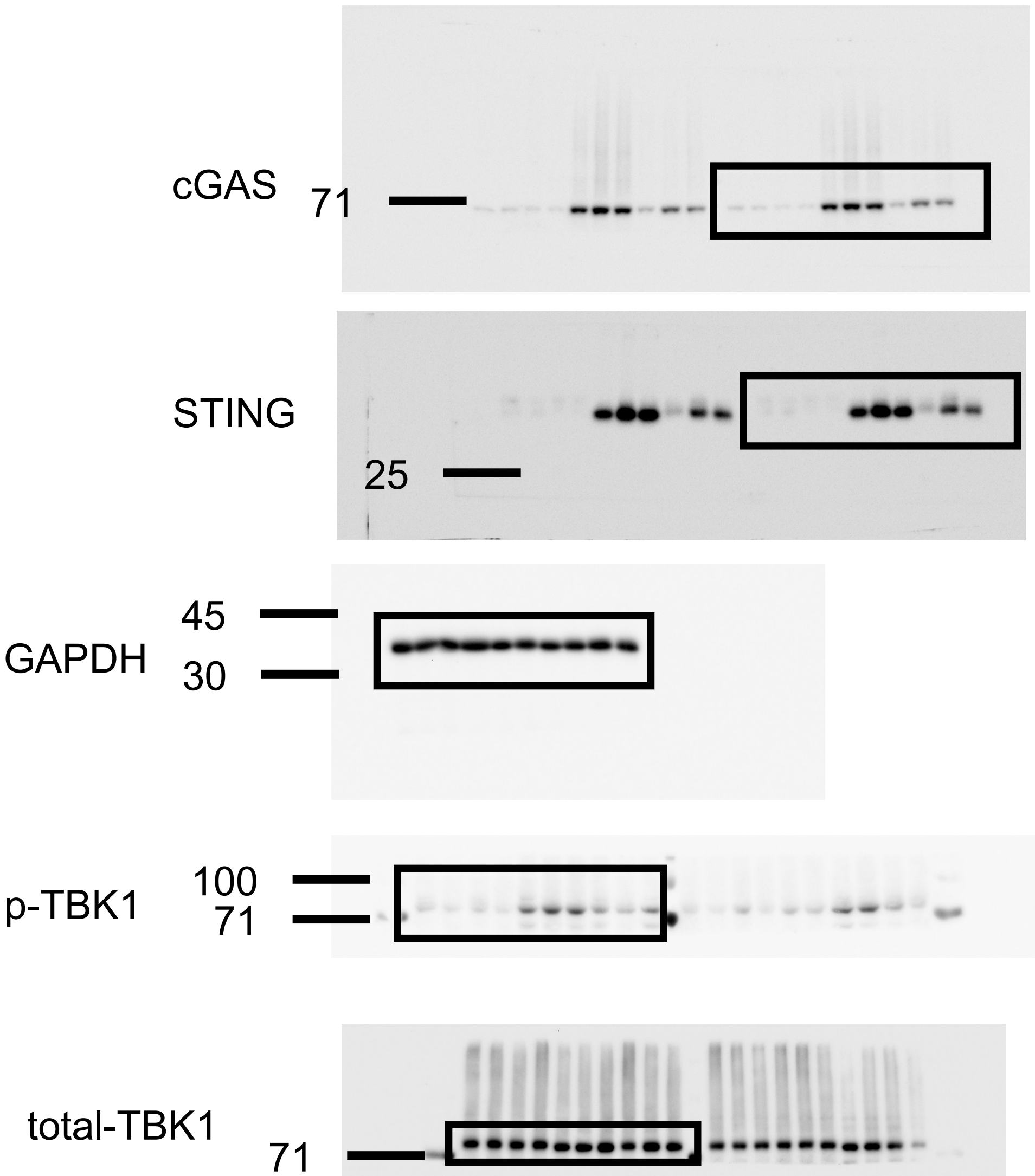

Supplementary Figure S8. Full Blots for Figure 5B

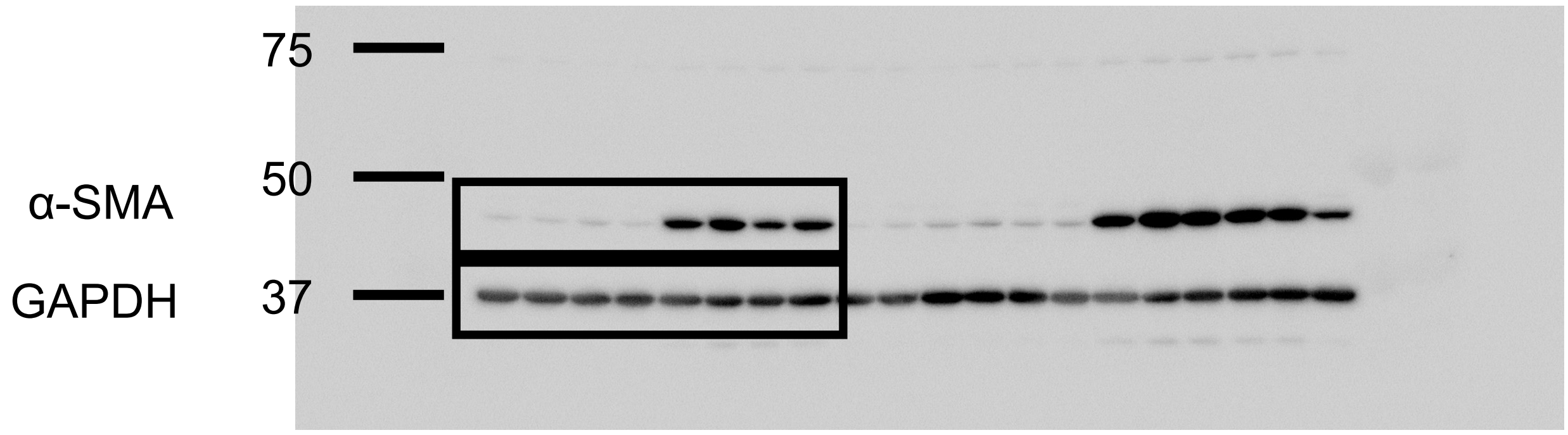

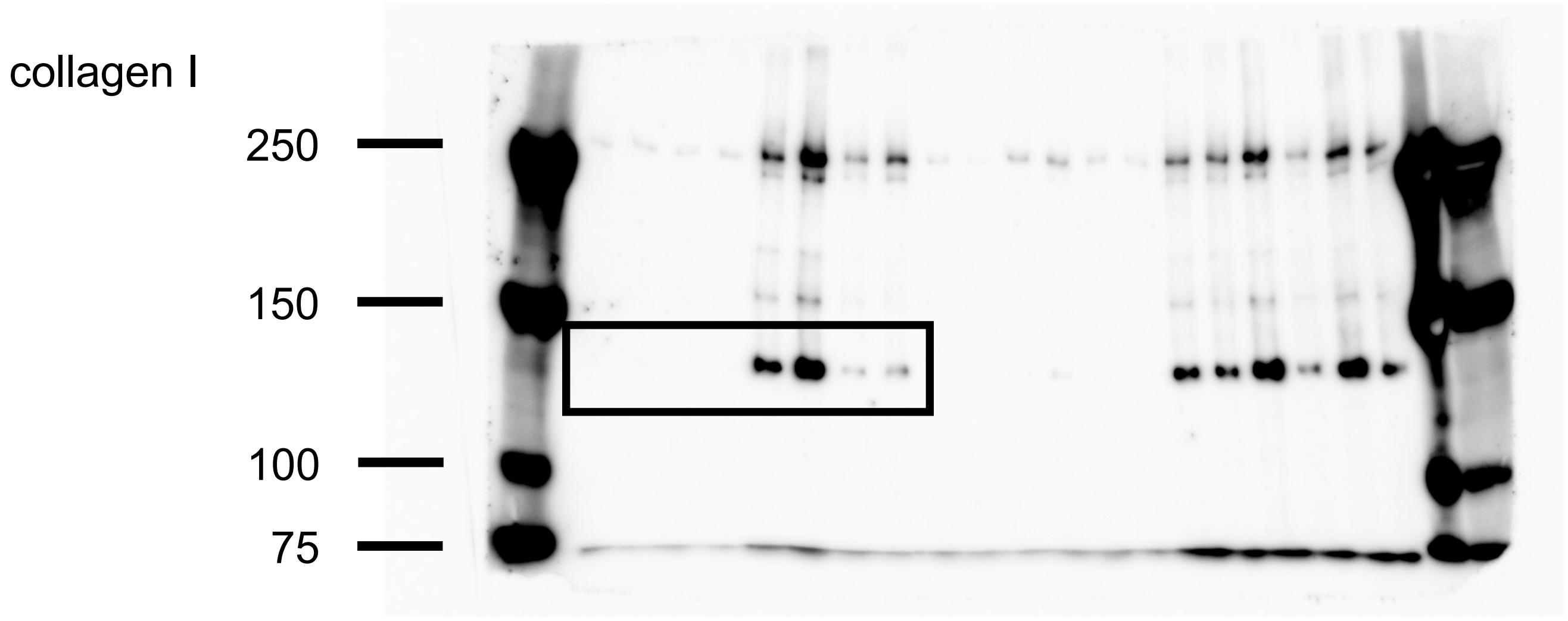

Supplementary Figure S9. Full Blots for Figure 6B

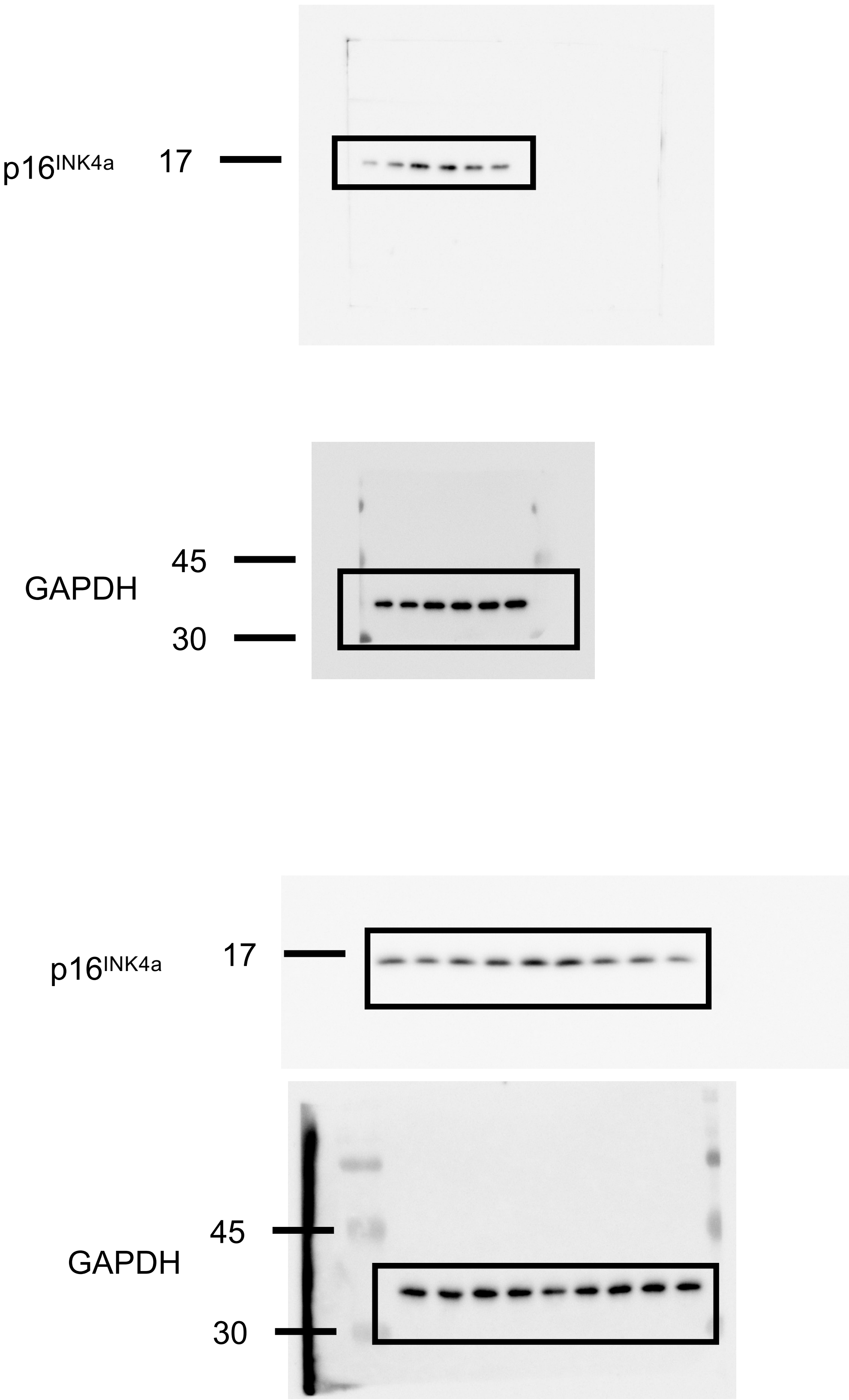

Supplementary Figure S10. Full Blots for Figure 7C, D

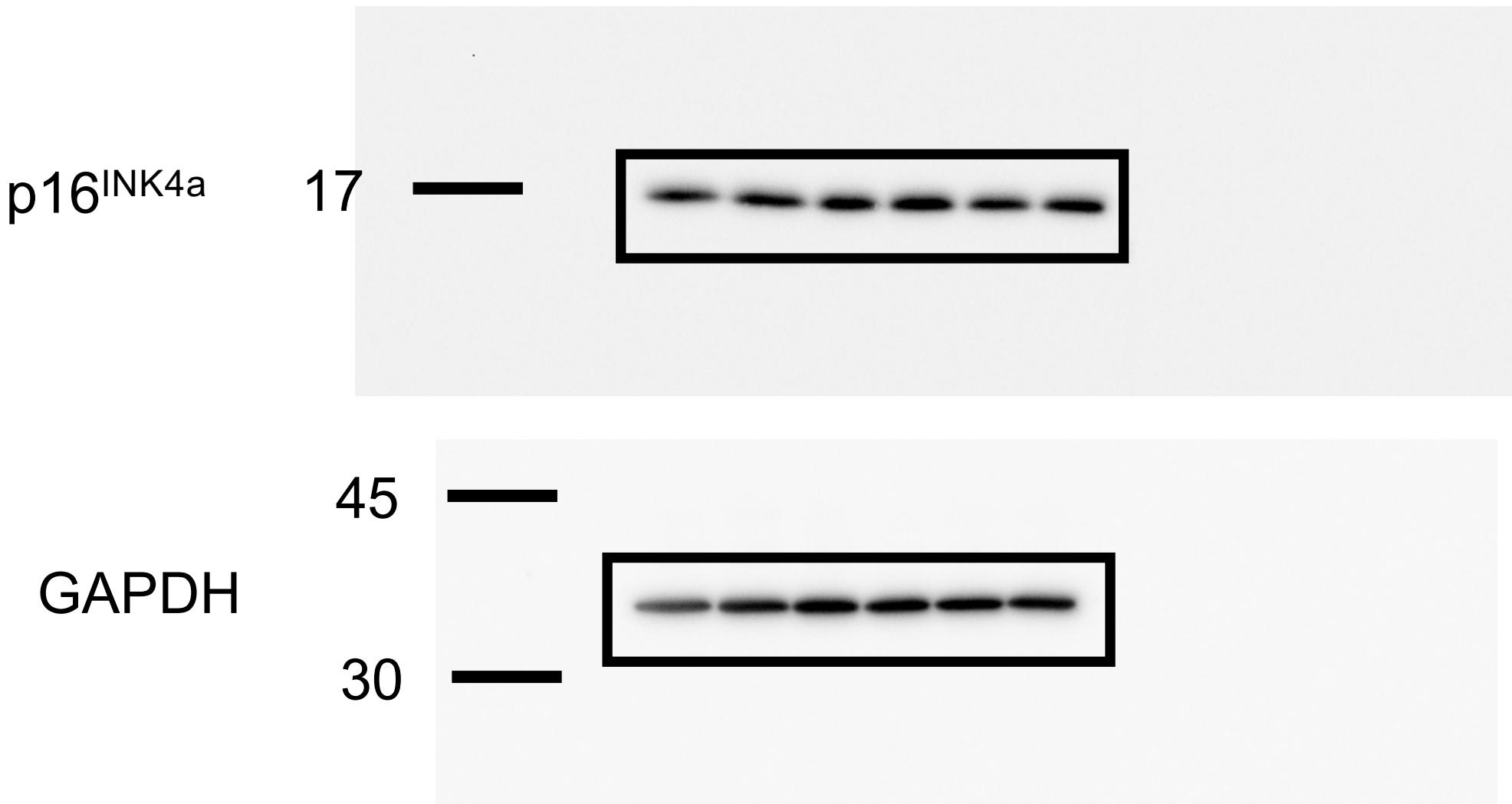

Supplementary Figure S11. Full Blots for Supplementary Figure S4
