# Supplementary material for: Magnesium Attenuates Renal Senescence and Fibrosis With Reduced DNA Damage Response and H3K4me3 Enrichment at the p16INK4a Promoter
Source: FASEB J. 2026 Aug 14;40(16):e72192. doi: 10.1096/fj.202504665RR (PMC13475955; doi:10.1096/fj.202504665RR)
Supplement: Supplementary file 2 [file FSB2-40-e72192-s001.docx]

**Supplementary Table S1. Primer sequences used for SYBR Green quantitative PCR analysis**

| **Gene** | **Forward primer (5′→3′)** | **Reverse primer (5′→3′)** | **Amplicon size (bp)** |
| --- | --- | --- | --- |
| *Tnf* | CCCTCACACTCAGATCATCTTCT | GCTACGACGTGGGCTACAG | 61 |
| *Ccl2* | TTAAAAACCTGGATCGGAACCAA | GCATTAGCTTCAGATTTACGGGT | 121 |
| *Cxcl1* | CTGGGATTCACCTCAAGAACATC | CAGGGTCAAGGCAAGCCTC | 117 |
| *Cxcl2* | CCAACCACCAGGCTACAGG | GCGTCACACTCAAGCTCTG | 108 |
| *Tgfb1* | CTCCCGTGGCTTCTAGTGC | GCCTTAGTTTGGACAGGATCTG | 133 |
| *Ccn2* | GGGCCTCTTCTGCGATTTC | ATCCAGGCAAGTGCATTGGTA | 151 |
| *Serpine1* | TTCAGCCCTTGCTTGCCTC | ACACTTTTACTCCGAAGTCGGT | 116 |
| *Ifnb1* | CAGCTCCAAGAAAGGACGAAC | GGCAGTGTAACTCTTCTGCAT | 138 |
| *Isg15* | GGTGTCCGTGACTAACTCCAT | TGGAAAGGGTAAGACCGTCCT | 131 |
| *Mx1* | GACCATAGGGGTCTTGACCAA | AGACTTGCTCTTTCTGAAAAGCC | 182 |
| *Cxcl10* | CCAAGTGCTGCCGTCATTTTC | GGCTCGCAGGGATGATTTCAA | 157 |
| *p16* | CGGTCGTACCCCGATTCAG | GCACCGTAGTTGAGCAGAAGAG | 72 |
| *Irf7* | GAGACTGGCTATTGGGGGAG | GACCGAAATGCTTCCAGGG | 102 |
| *Ifit1* | CTGAGATGTCACTTCACATGGAA | GTGCATCCCCAATGGGTTCT | 117 |
| *Gapdh* | AGGTCGGTGTGAACGGATTTG | TGTAGACCATGTAGTTGAGGTCA | 123 |
| *Rps18* | AGTTCCAGCACATTTTGCGAG | TCATCCTCCGTGAGTTCTCCA | 166 |
